# Effect of pharmacist interventions in chronic kidney disease: a meta-analysis

**DOI:** 10.1093/ndt/gfae221

**Published:** 2024-10-09

**Authors:** Ashkon Ardavani, Ffion Curtis, Ellen Hopwood, Patrick Highton, Priscilla Katapa, Kamlesh Khunti, Thomas J Wilkinson

**Affiliations:** NIHR Applied Research Collaboration East Midlands (ARC-EM), Leicester Diabetes Centre, University of Leicester, Leicester, UK; Liverpool Reviews and Implementation Group (LRIG), Institute of Population Health, University of Liverpool, Liverpool, UK; NIHR Applied Research Collaboration East Midlands (ARC-EM), Leicester Diabetes Centre, University of Leicester, Leicester, UK; NIHR Applied Research Collaboration East Midlands (ARC-EM), Leicester Diabetes Centre, University of Leicester, Leicester, UK; NIHR Applied Research Collaboration East Midlands (ARC-EM), Leicester Diabetes Centre, University of Leicester, Leicester, UK; NIHR Applied Research Collaboration East Midlands (ARC-EM), Leicester Diabetes Centre, University of Leicester, Leicester, UK; NIHR Leicester Biomedical Research Centre (BRC), Leicester Diabetes Centre, University of Leicester, Leicester, UK

**Keywords:** chronic kidney disease, meta-analysis, outcomes, pharmacist, systematic review

## Abstract

**Background:**

Pharmacists are uniquely placed with their therapeutic knowledge to manage people with chronic kidney disease (CKD). Data are limited regarding the impact of pharmacist interventions on economic, clinical and humanistic outcomes (ECHO).

**Methods:**

A systematic review and meta-analysis of randomized controlled trials (RCTs) of interventions with pharmacist input was conducted, which included adults with a diagnosis of CKD, including those with and without kidney replacement therapy. Data were extracted on ECHO: economic (e.g. healthcare-associated costs), clinical (e.g. mortality) and humanistic (e.g. patient satisfaction) outcomes. Where appropriate, a random-effects model meta-analysis generated a pooled estimate of effect. A direction of effect plot was used to summarize the overall effects for clinical outcome domains.

**Results:**

Thirty-two RCTs reported a total of 10 economic, 211 clinical and 18 humanistic outcomes. Pharmacist interventions resulted in statistically significant improvements in systolic blood pressure and hemoglobin levels, but not in diastolic blood pressure, estimated glomerular filtration rate, creatinine and low-density lipoprotein cholesterol levels. Mixed findings were reported for clinical and economic outcomes, whilst pharmacist interventions resulted in an improvement in humanistic outcomes such as patient satisfaction and patient knowledge.

**Conclusion:**

Findings showed pharmacist interventions had mixed results for various outcomes. Future studies should be more robustly designed and take into consideration the role of the pharmacist in prescribing and deprescribing, the findings of which will help inform research and clinical practice.

**Trial registration:**

The review was prospectively registered on PROSPERO (CRD42022304902).

## Graphical Abstract

**Graphical Abstract:**
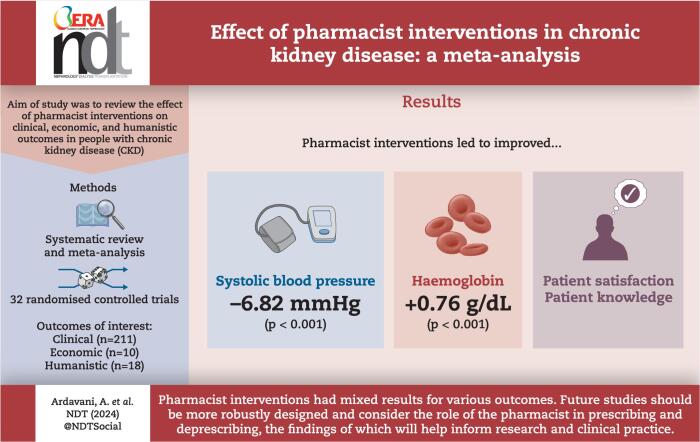

KEY LEARNING POINTS **What was known:**

- Chronic kidney disease (CKD) is a progressive condition associated with various comorbidities and complications, and demanding complex medical and medicine management.
- Pharmacists are well positioned within the healthcare system to help manage and support people living with CKD.
- Currently, data are limited concerning the impact of pharmacist interventions on economic, clinical and humanistic outcomes.

**This study adds:**

- Pharmacist interventions resulted in a statistically significant improvement in systolic blood pressure and hemoglobin levels.
- No statistically significant improvements were reported for diastolic blood pressure, estimated glomerular filtration rate, low-density lipoprotein cholesterol and creatinine levels.
- Mixed findings were reported clinical and economic outcomes, although pharmacist interventions did result in an improvement in humanistic outcomes such as patient satisfaction.

**Potential impact:**

- Pharmacists can have a significant impact on the health of people living with CKD with improvements in outcomes likely driven by better pharmacological management.
- Randomized controlled trials (RCTs) should be more robustly designed, incorporating intention-to-treat analysis, and exploring the impact of pharmacist prescribing and deprescribing.
- Future adaptive RCTs are needed to identify what aspects of a multicomponent intervention delivered by pharmacists are most effective.

## INTRODUCTION

Pharmacists are well positioned within the healthcare team to manage people with chronic kidney disease (CKD). Clinical interventions delivered by pharmacists are highly varied and include, but are not limited to, managing complications such as anemia, medication management including dose adjustment, and delivering patient education [1]. In primary care, where the majority of patients are managed [2], pharmacists are in an optimal position to provide targeted screening and implement early interventions [3].

Previous research has illustrated the benefits of pharmacist interventions in CKD. For example, pharmacist-led medication reconciliation reduced medication discrepancies and medication-related problems [4] and pharmacist-led education sessions increased post-transplant graft survival and improved management of immunosuppressants [5]. However, pharmacists remain underutilized members of many CKD multidisciplinary teams [6]. Several factors may be attributed to this, including insufficient time for practicing clinically, inadequate cover for services [7], lack of available resources to enable development as prescribers, and a scarcity of evidence-informed recommendations. With the global burden of CKD increasing and 96% of CKD patients having additional multiple long-term conditions [8, 9], pharmacists continue to provide an increasingly valuable contribution towards CKD management [8]. The emergence of pharmacological therapies, such as sodium-glucose cotransporter-2 inhibitors (SGLT2Is) [10] and emphasis on patient self-management/education [11], means that pharmacists are central to ensuring the provision of safe and effective treatment [10], particularly as patients are likely to increasingly rely on care provided by pharmacists as part of multi-professional teams across primary care networks [12]. Strengthening the evidence base around the effect of the pharmacist will help optimize their role, identify interventions they are best placed to deliver and reduce the burden on healthcare systems.

Data on the extent to which pharmacist interventions result in an improvement in economic, clinical and humanistic outcomes (ECHO) is limited. Whilst several systematic reviews have explored the impact of pharmacist interventions in CKD [13–15], they are restricted due to heterogeneity in research study designs and narrow conceptual search terms. With these reviews now out of date, the proliferation of new pharmacological technologies, changes in healthcare systems post-COVID-19 and new research available [16, 17], a contemporary and more comprehensive evidence synthesis is required. The aims of this review were to (i) establish the effect of pharmacist interventions on ECHO in CKD, and (ii) identify the structures and processes of effective interventions.

## MATERIALS AND METHODS

### Registration and reporting

The review was prospectively registered on the International Prospective Register of Systematic Reviews (PROSPERO) (CRD42022304902) and a detailed protocol prospectively has been published [18]. The review is reported in accordance with the Preferred Reporting Items for Systematic Reviews and Meta-Analyses (PRISMA) 2020 statement (Supplementary data, Table S1) [19].

### Eligibility criteria

Inclusion and exclusion criteria can be found in Supplementary data, Table S2.

### Information sources

Bibliographic databases (MEDLINE, Scopus and Web of Science) were searched from inception to February 2023. The Cochrane Library and PROSPERO were searched for existing and ongoing reviews. Database searches were supplemented with internet searches (i.e. Google Scholar) and forward and backward citation tracking from included studies and review articles. See Supplementary data, Table S3 for an example search strategy. Search terms were developed with library support at the University of Leicester.

### Selection process

Results from searches were combined in EndNote 20 (Clarivate Analytics), where duplicates were removed before uploading retrieved references to Covidence [20]. Two reviewers independently screened titles and abstracts for inclusion. Discrepancies were resolved through discussion or with input from a third reviewer. Full texts were obtained for eligible reports and independently screened by two reviewers.

### Data collection process

A bespoke extraction form was created to document extracted information from the studies. The form was piloted and amended before full extraction. One reviewer extracted data and a second checked for accuracy.

### Data items

The extracted information included the following: sociodemographic (e.g. age, sex, ethnicity) and clinical characteristics (e.g. blood pressure), details of the intervention and control groups such as sample size, the type of intervention and duration, and all outcomes as reported in primary studies. Information was extracted on the resources available for pharmacists for provision of care and the processes they carried out.

### Outcomes

Data was extracted based on the ECHO model: economic (e.g. hospitalization-associated costs), clinical (e.g. mortality) and humanistic [e.g. health-related quality of life (HRQoL)].

### Quality assessment

Two reviewers independently assessed the study quality and risk of bias using the United States National Heart Lung and Blood Institute quality assessment tool for controlled intervention studies [21]. Studies were ranked as “good,” “fair” or “poor” based on criteria found in Supplementary data, Table S4.

### Data synthesis

A narrative synthesis was created from the included studies structured around the type of intervention, target population characteristics and outcome measures. Data were presented using text, tables, diagrams and an effect direction plot [22]. If the direction of effect for multiple outcomes across studies for an umbrella outcome was the same, then that effect direction was reported [22]. However, if there was variation across studies, then the direction of effect was reported if the majority (≥70%) reported a similar direction; a sideways arrow was used if <70% of outcomes reported a constant direction of effect [22]. Where appropriate, data were pooled using a meta-analysis to generate an overall measure of effect using RevMan Web (The Cochrane Collaboration). Continuous data was expressed as a mean or standardized mean difference with a 95% confidence interval (CI). A random-effects model was used for the meta-analysis [23]. Data were not pooled if heterogeneity was moderate (*I*^2^ statistic >40%) [24]. The level of significance was set at <.05. Potential causes of heterogeneity were explored in analyses with an *I*^2^ statistic >40%. A sensitivity analysis was performed using the “one-study-removed” procedure [25]. This process was repeated until the I^2^ value fell <40%. If more than one study went below this threshold, the study that resulted in the largest reduction in the I^2^ value was omitted [25]. If this approach did not cause the *I*^2^ value to become <40%, then “poor” quality studies were removed [26].

### Patient consent

This review did not require patient recruitment and did not require patient consent.

## RESULTS

### Study selection

**Figure 1:**
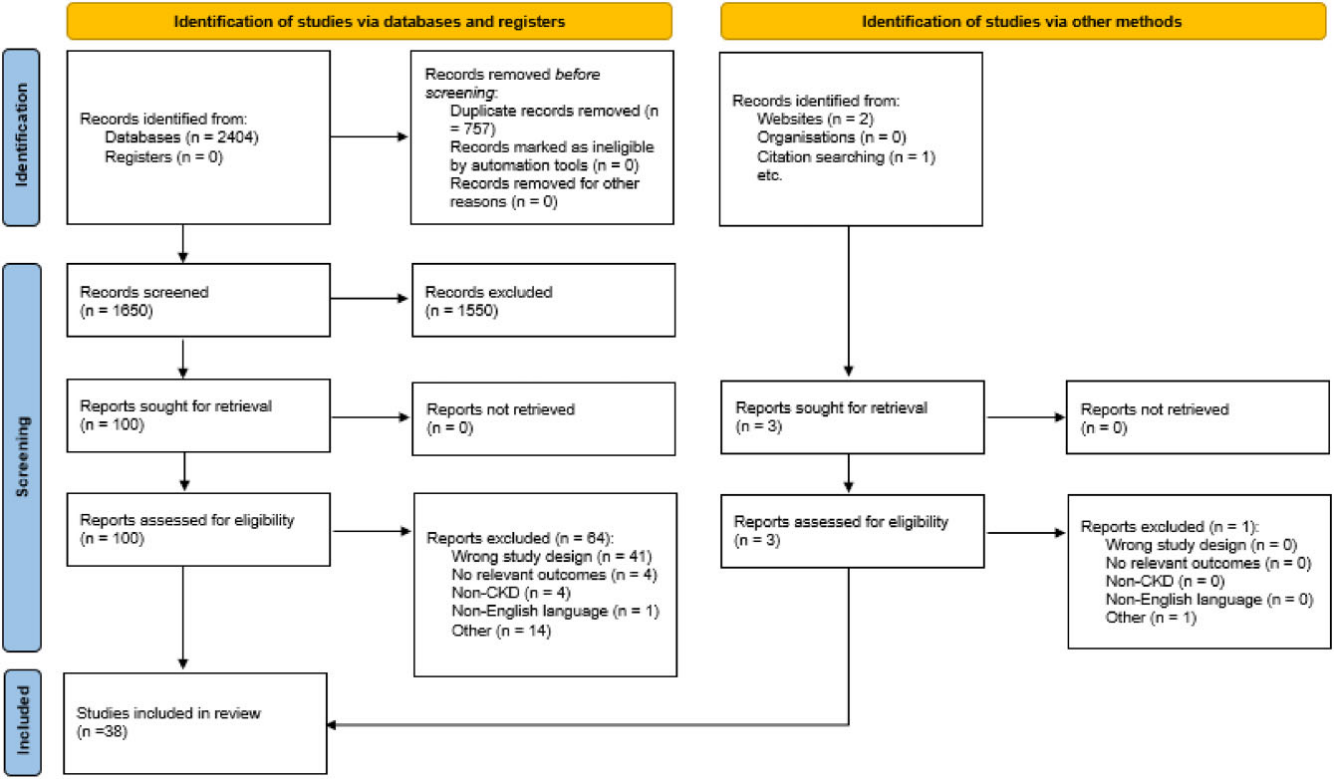
PRISMA flow diagram.

A total of 2407 references were identified, and following the removal of duplicates and screening of full texts for eligibility assessment a total 38 references were included (Fig. 1). These 38 reports were based on 32 randomized controlled trials (RCTs), as several reported data on different outcomes from the same RCTs; these included Fleming *et al.* [27–29] (TRANSAFE Rx study), Mateti *et al.* [30–32], Lalonde *et al.* [33, 34] and Pai *et al.* [35, 36].

### Study characteristics

A summary of the characteristics of included studies can be found in Table 1. The 32 RCTs were conducted in 12 countries across five continents: the USA (*n* = 16), Canada (*n* = 3), India (*n* = 3), Jordan (*n* = 2), Brazil (*n* = 1), Iran (*n* = 1), Iraq (*n* = 1), the Netherlands (*n* = 1), Nigeria (*n* = 1), South Korea (*n* = 1), Thailand (*n* = 1) and the UK (*n* = 1). The total sample size of the RCTs ranged from 24 to 32 917 participants. Where reported, the mean age of participants in the control and intervention groups ranged from 43.1 to 75.7 years and 45.7 to 75.6 years, respectively; two studies did not report age [37, 38]. The study populations were heterogenous. Twelve (38%) studies were conducted in those undergoing dialysis, 6 (19%) in kidney transplant recipients, 13 (41%) in those with non-dialysis dependent CKD (stages 1–5) and 1 (3%) study in a mixed (dialysis and non-dialysis) cohort. The RCTs were conducted in various settings, five (16%) in primary care such as clinics and community pharmacies, 18 (56%) in secondary care (e.g. hemodialysis centers, post-transplant clinics), seven (22%) in tertiary care (e.g. dialysis units at tertiary hospitals) and two (6%) studies were conducted in both primary and secondary care settings. Structures and processes were reported in detail and can be found in Table 1.

**Table 1:** Characteristics of included studies.

First author (year), country [reference]	Sample size	Age (years)	Population (eGFR if reported)	Summary of structures	Summary of pharmacist (involvement in) interventions	Summary of outcomes (ECHO model)
Al Hamarneh (2018), Canada [58]	Total: 290; control: 147; intervention: 143	Control: 61.0 ± 11.8; intervention: 61.3 ± 12.4	CKD (eGFR: control: 63.9 ± 26.1; intervention: 65.5 ± 25.7)	Pharmacist training program, developed by the research team based on Canadian guidelines, and documentation of care plans for remuneration; intervention delivered only by a pharmacist	Patient assessments, laboratory assessments, individualized CV risk assessment, therapeutic recommendations, prescription adaptation(s) and/or initiation, and regular monthly follow-up	Economic: N/RClinical: estimated CV risk, LDL, SBP, DBP, HbA1C, smoking cessation, 5-year predicted risk for developing ESKD, initiation of ACEI, statins and ARBs, dose changes and medication changes for diabetes, dyslipidemia, hypertension, and impact of rural vs urban residence on the difference in change in estimated CV risk
						Humanistic: N/R
Alshogran (2022), Jordan [16]	Total: 120; control: 60; intervention: 60	Control: 51.6 ± 16; intervention: 50.9 ± 17	HD	An instructional booklet that was developed using available ESKD-related guidelines; the booklet was verified by two nephrologists to ensure it was accurate; intervention delivered by pharmacist, in addition to usual care	Patient education on disease management and complications, medication information and adherence, potential adverse drug events, diet selection, the importance of adhering to dialysis management, diet and fluid restrictions, the importance of regular follow-up with the medical team, completing required laboratory testing and exercising	Economic: N/RClinical: patients’ total adherence to recommendations, HD attendance adherence, diet restriction adherence, duration of shortening HD, episodes of shortening HD, fluid restriction adherence, medication adherence, hospital and emergency room admissions after follow-up, and levels of sodium, potassium, urea, creatinine, albumin, calcium, corrected calcium, phosphorus, calcium*phosphorus product, hemoglobin and PTH
						Humanistic: patients’ perception of adherence behaviors, disease awareness and HRQoL
Armstrong (2000), USA [39]	Total: 566; control: 278; intervention: 288	Control: 57.2 ± 16.1; intervention: 52.2 ± 14.6	HD	A 4-day training session for pharmacists for managing patients undergoing long term hemodialysis, use of the EPOCALC™ software program, and review of the specific DUE criteria; intervention involved trained pharmacist worked in collaboration with a nephrologist	In collaboration with the nephrologist, the pharmacist monitored, evaluated and recommended initial and subsequent medication dosage regimens	Economic: N/RClinical: serum ferritin and/or transferrin saturation, hematocrit measurements, adverse events, effective treatment of anemiaHumanistic: N/R
Bessa (2016), Brazil [40]	Total: 128; control: 64; intervention: 64	Control: 43.1 ± 12.5; intervention: 45.7 ± 11.6	KTR	Visual aids and a checklist; interventions delivered by five pharmacists	MR, patient education on medications, diet, and physical activity, and patient counselling on medication use	Economic: N/RClinical: %CV, mean %CV, % of patients who achieved TAC target concentrations in each study visit, comparison of mean dose-corrected whole blood TAC trough concentrations from day 7 to day 90, incidence of infections, acute rejection, eGFR, death, graft loss, discontinuation of immunosuppressive treatment, hospital readmissions, medication adherenceHumanistic: N/R
Bhardwaja (2011), USA [54]	Total: 32 917; control: 16 340 intervention: 16 577	Control: 77 (56–91); intervention: 77 (57–91)^c^	CKD	DRAP program, PIMS and medication decision guide; intervention delivered by the pharmacist	Pharmacist used medication guide to inform physician on drugs that require dose adjustment or should be avoided, and patients were counselled on rationale for drug changes	Economic: N/RClinical: proportion of medication errorsHumanistic: N/R
Chang (2016), USA [41]	Total: 47; control: 23; intervention: 24	Control: 70.6 (9.7); intervention: 64.0 (13.2)^a^	CKD [eGFR: control: 53.8 (5.8); intervention: 54.1 (7.7)^a^]	Training for pharmacist on comorbidities like dyslipidemia, diabetes and hypertension, *JAMA Hypertension* 2014 guidelines; pharmacist was educated by a nephrologist, KDIGO 2012 Clinical Practice Guideline for the Evaluation and Management of Chronic Kidney Diseases; intervention delivered by pharmacists	Review charts, order lipid and UACR screening tests, manage BP and lipid therapy according to KDIGO guidelines. Participants contacted by phone if proteinuria screening needed to be completed, and if needed, medication initiation and/or titration	Economic: N/RClinical: proteinuria screening and lipid screening within 1 year of the enrolment date, achieved BP goal (<140/90 mmHg for non-proteinuric CKD and <130/80 mmHg for proteinuric CKD), proportion of proteinuric CKD patients taking an ACEI or ARB, proportion of patients on statinsHumanistic: patient survey on acceptability of pharmacist MTM
Chisholm-Burns (2013), USA [59]	Total: 150; control: 74; intervention: 76	Control: 51.3 ± 13.7; intervention: 52.8 ± 13.6	KTR	Clinical pharmacist trained by lead investigator on the contracting process, toolbox of standardized solutions to adherence barriers, IST adherence contracts generally followed format described by Haber and Rhodes, pharmacy refill records were obtained from Avella Specialty Pharmacy, direct medical costs were estimated using the 2009 Medicare Expenditure Panel Survey, US mail was used for administering healthcare screening questionnaire; clinical pharmacist conducted all intervention activities	Discussion with patient regarding components of a renal transplant recipient adherence contract to achieve highest possible immunosuppressant adherence	Economic: cost-savingClinical: immunosuppressant adherence, days in hospital, emergency department visits, outpatient visits and homecare visitsHumanistic: N/R
Chisholm (2001), USA [37]	Total: 24; control: 12; intervention: 12	N/R for each group; however, the mean age of cohort was 49.2 ± 10.2 years	KTR	Patients’ medical records and the MCG Outpatient Pharmacy's prescription computer system (National Data Corporation^©^) was used to collate data from patients’ pharmacy refill records; intervention delivered by a pharmacist	Medicine optimization, recommendations to nephrologists, patient counselling on medication use, medication reviews and histories, and promotion of medication compliance	Economic: N/RClinical: immunosuppressant compliance rates, patterns of compliance and serum immunosuppressant concentrations
Chisholm (2002), USA [42]	Total: 26; control: 12; intervention: 14	Control: 47 ± 12.7; intervention: 51 ± 16.8	KTR	N/R; intervention delivered by a clinical pharmacist	Medication recommendations to nephrologist, patient counselling for medication(s) and medication reviews and histories	Economic: N/RClinical: SBP and DBP
Cohen (2020), USA [43]	Total: 200; control: 100; intervention: 100	Control: 58 (25.7–82.9); intervention: 54.4 (20.8–76.2)^e^	KTR	EMR medication lists and patient-reported medication lists from patients’ outpatient pharmacy; intervention delivered solely by pharmacist	Medication history, MR, information sharing with primary care team and patient's pharmacy and telephone audits	Economic: N/RClinical: % of patients with inadequate MR determined by any one error in MR, the number of medication errors, of all medications and high-risk medications, identified per patient sampleHumanistic: N/R
Cooney (2015), USA [60]	Total: 2199; control: 1129; intervention: 1070	Control: 75.7 (8.2); intervention: 75.6 (8.2)^a^	CKD [eGFR: control: 34.5 (7.3); intervention: 34.2 (7.7)^a^]	CKD registry, EHR, standard approach to managing CKD based on recommendations from KDOQI guidelines was developed and reviewed with study pharmacists and a CKD informational packet from the National Kidney Disease Education Program (Chronic Kidney Disease—What Does it Mean for Me?); intervention delivered by two pharmacists as part of a pharmacist–physician collaboration	Phone-based pharmacist intervention, where the pharmacist reviewed medications and lifestyle modifications with patients, ordering KDOQI recommended labs, ordering nephrology results for patients with severe eGFR, follow up phone call to review of any abnormal lab results and initiate appropriate medication changes when required, pharmacist-physician collaboration, and patient education	Economic: N/RClinical: SBP among participants with baseline BP >130/80 mmHg, PTH measured during study period, BP <130/80 mmHg, incidence of ESKD, death, measurement of phosphorus and UACR; the number of antihypertensive medications prescribed to those with poorly controlled hypertension; appropriate treatment with ACEI/ARB, phosphorus binders, vitamin D, and sodium bicarbonate; medication adherence; and the percent of subjects seen by nephrologyHumanistic: HRQoL and patient satisfaction
Cypes (2021), USA [44]	Total: 182; control: 87; intervention: 95	Control: 76 (69–81); intervention: 77 (70–85)^b^	CKD [eGFR: control: 40 (31–53); intervention: 37 (28–46)^b^]	EHRs and medical charts; intervention delivered as part of an interprofessional team	Medication recommendations for identification and resolution of medication-dosing errors and improved collaboration between pharmacist and providers	Economic: N/RClinical: number of medications requiring pharmacist intervention and incorrect CKD stagingHumanistic: N/R
Dashti-Khavidaki (2013), Iran [45]	Total: 92; control: 47; intervention: 45	Control: 48.6 ± 14.7; intervention: 55.4 ± 15.7	HD	An investigator-designed form for collecting patients’ demographic, clinical and laboratory data, education by an expert psychologist, patients’ charts and prescriptions, and two booklets regarding correct drug administration and nutrition; intervention delivered as part of a collaboration	Evaluation of medication adherence and identification of DRPs, dose adjustment, patient education about disease, medications, lifestyle modification and nutrition, nutritional consultation, and motivational interviewing	Economic: N/RClinical: N/RHumanistic: HRQoL
Fleming (2021), USA [27]	Total: 136; control: 68; intervention: 68	Control: 51.2 (13.7); intervention: 50.2 (12.3)^a^	KTR	A smartphone-enabled mobile health app and an accurate list of patients’ medication regimen, which was automatically updated from the EMR; intervention delivered by a clinical transplant pharmacist	Clinical pharmacist– led supplemental medication therapy monitoring and management, utilizing a smartphone-enabled mobile health app, integrated with risk-driven televisits and home-based BP and blood glucose monitoring (when applicable), telemonitoring of medications, medical appointment adherence, weekly BP/glucose readings, and scheduling telehealth visits with participants	Economic: N/RClinical: mean TAC intrapatient variability from baseline to 12 months post randomization and proportion of patients achieving TAC intrapatient variability of <30% and <40% at end of studyHumanistic: patient feedback on app
Gonzales (2021), USA [28]	Total: 136; control: 68; intervention: 68	Control: 51 ± 13; intervention: 50 ± 12	KTR	A smartphone-enabled mobile health app and an accurate list of patients’ medication regimen, which was automatically updated from the EMR; intervention delivered by a clinical transplant pharmacist	Clinical pharmacist– led supplemental medication therapy monitoring and management, utilizing a smartphone-enabled mobile health app, integrated with risk-driven televisits and home-based BP and blood glucose monitoring (when applicable), telemonitoring of medications, medical appointment adherence, weekly BP/glucose readings, and scheduling telehealth visits with participants	Economic: N/RClinical: incidence and severity of medication errors, incidence and severity of adverse events, adverse event rate, hospitalization rate, infection rate and opportunistic infection rateHumanistic: N/R
Ishani (2016), USA [55]	Total: 601; usual care: 150; intervention: 451	Usual care: 74.3 ± 8.1; intervention: 75.3 ± 8.1	CKD (eGFR: usual care: 38 ± 8; intervention: 37 ± 9)	KDIGO 2012 Clinical Practice Guideline for the Evaluation and Management of Chronic Kidney Disease, components of the chronic care model, and customized education program delivered over broadband; intervention delivered as part of a collaboration	Management of CKD and comorbidities, lifestyle modifications, development of a customized education program, and medication management	Economic: N/RClinical: a composite of death, hospitalization, emergency department visits, and admission to a skilled nursing facility, each component of the composite, and incidence of ESKDHumanistic: N/R
Lalonde (2017), Canada [33]	Total: 442; control: 138; ProFil: 304	Control: 71.2 ± 12.5; ProFil: 71.9 ± 12.0	CKD (eGFR: usual care: 28.2 ± 10.7; ProFil: 26.8 ± 9.3)	90-min interactive web-based training program medication use in CKD, a clinical guide, patients’ clinical summaries, facilitated access to the CKD clinic, pharmaceutical opinion form, CKD clinic chart, and pharmacy medication renewal chart; intervention delivered by community pharmacists as part of a collaborative and multidisciplinary care program	Prevention, detection, and management of DRPs using pharmaceutical opinion form and communication with CKD pharmacist regarding queries	Economic: N/RClinical: mean number of DRPs, eGFR, SBP, DBP, HbA1C, LDL cholesterolHumanistic: N/R
Marouf (2020), Iraq [46]	Total: 120; control: 60; intervention: 60	Control: 51.6 ± 17.8; intervention: 49.4 ± 14.6	HD	International guidelines for treating anemia in CKD and Microsoft Excel; intervention delivered by pharmacist as part of pharmacist–physician collaboration	Pharmacist–physician collaboration for developing an in-hospital guideline for the proper use of recombinant human erythropoietin, provision of drug information on CKD-associated anemia to physicians and nurses; involvement included: ordering laboratory tests; medication adjustments, education/counselling, and development of management plans	Economic: N/RClinical: hemoglobin, TSAT, serum ferritin, serum vitamin B12, serum folate and clinical grading of pallorHumanistic: N/R
Mateti (2017, 2018), India [30–32]	Total: 200; academic hospital (usual care: 52; pharmaceutical care: 52); government hospital (usual care: 13; pharmaceutical care: 13); corporate hospital (usual care: 35; pharmaceutical care: 35)	Academic hospital (usual care: 49.4 ± 12.5; pharmaceutical care: 52.8 ± 10.5); government hospital (usual care: 48 ± 17 years; pharmaceutical care: 49.2 ± 12.6); corporate hospital (usual care: 53.8 ± 11.9; pharmaceutical care: 53.0 ± 15.1)	HD	Validated pictogram-based information leaflets and validated protocols, and a customized care plan based on WHO-FIP Pharmaceutical care model; intervention consisted of usual care (by hospital staff such as physicians, nurses, and technicians) along with pharmaceutical care delivered by a qualified registered pharmacist	Patient education and motivation, with validated protocols regarding knowledge about disease, medication, lifestyle changes, nutritional information, personal interview and medication review. Nutritional advice for HD and comorbidities, education on foodstuffs containing potassium, phosphate, protein, sodium, depending on the patients’ prerequisite, and fluid constraints, advice on medication administration, laboratory monitoring and adherence to HD and medication issues	Economic: utility values and QALY, cost data, cost-effectiveness grid and plane and ICERClinical: SBP, DBP, IDW, hemoglobin levels, survival time and medication adherence rateHumanistic: HRQoL
Okoro (2022), Nigeria [17]	Total: 147; usual care: 74; intervention: 73	Usual care: 53.31 ± 12.1; pharmacists’ intervention: 51.08 ± 13.4	CKD	CKD patient educational infographic leaflet, a calibrated sphygmomanometer digital BP monitor, BP logbook, hands-on training on BP self-measurement, and mobile phone; interventions delivered by pharmacists	Face-to-face group education on CKD, dietary recommendations, hypertension as a comorbidity and its management; counselling to improve antihypertensive medication adherence; individualized prescription review; antihypertensive medication adherence and HBPM reminder cell phone text messages; drug-related interventions; phone-in inquiries from the participants, and phone-out reinforcement interventions	Economic: N/RClinical: changes in BP from baseline to 6 months and 12 months, from 6 to 12 months, proportion of participants with controlled BP to less than 130/80 mmHg at 6 and 12 months, respectively; changes in antihypertensive medication adherence and serum creatinine levels from baseline to 6 and 12 months, and 6 to 12 months, respectivelyHumanistic: patients’ satisfaction with care received
Pai (2009), USA [35]	Total: 107; standard of care: 46; pharmaceutical care: 61	Standard of care: 60 ± 15; pharmaceutical care: 55.8 ± 15.1	HD	Laboratory data, drug chart, postdoctoral training in nephrology pharmacotherapy, and Hepler and Strand classification of drug-related problems; intervention delivered by a nephrology-trained clinical pharmacist or one of two pharmacists completing postdoctoral training in nephrology pharmacotherapy	One on one patient interview, medication review—generation of a current medication profile, identification, and addressing various DRPs, provision of healthcare provider and patient education, provision of cognitive services (patient-oriented services that focused on optimizing drug therapy) and formal reviews of the patients with the multidisciplinary healthcare team	Economic: N/RClinical: N/RHumanistic: HRQoL
Pai (2009), USA [36]	Total: 104; standard of care: 47; pharmaceutical care: 57	Standard of care: 60.5 ± 14.7; pharmaceutical care: 56.3 ± 15	HD	Laboratory data, drug chart, postdoctoral training in nephrology pharmacotherapy, and Hepler and Strand classification of drug-related problems; intervention delivered by a nephrology-trained clinical pharmacist or one of two pharmacists completing postdoctoral training in nephrology pharmacotherapy	One on one patient interview, medication review—generation of a current medication profile, identification, and addressing various DRPs, provision of healthcare provider and patient education, provision of cognitive services (patient-oriented services that focused on optimizing drug therapy) and formal reviews of the patients with the multidisciplinary healthcare team	Economic: cost of all concomitant drugsClinical: number of medications used, rate of hospitalization, length of hospitalizationHumanistic: N/R
Peralta (2020), USA [61]	Total: 524; usual care: 188; ECDSS: 165; ECDSS Plus: 171	Usual care: 71.1 ± 8.4; eCDSS: 70.2 ± 8.6; eCDSS-PLUS: 69.4 ± 9.6	CKD (eGFR: usual care: 56 ± 12.2; eCDSS: 55 ± 11.5; eCDSS-PLUS: 58 ± 11.4)	Educational materials, based on National Kidney Disease Education Program Materials, and EHRs; intervention delivered as part of a collaboration	Phone call to reinforce medication changes ordered at the PCP visit, CKD-related teaching and a comprehensive medication review	Economic: N/RClinical: SBP, DBP, controlled BP (<140/90 mm Hg), ACEi/ARB use, ACEi/ARB initiation, statin therapy use, statin therapy initiation, diuretic use and diuretic initiationHumanistic: N/R
Qudah (2016), Jordan [47]	Total: 56; control: 27; intervention: 29	Control: 51.7 ± 18.5; intervention: 55.3 ± 15.1	HD	KDOQI WorkGroup 2005 guidelines, BP monitors, and logbook of BP readings; intervention delivered as part of a pharmacist–physician collaboration	Pharmacist–physician collaboration to optimize antihypertensive therapy, provision of educational materials, patient counselling for adherence to antihypertensive medication, fluid and salt restrictions, dialysis sessions, and patient education and explanation for goals for BP and daily weight gain	Economic: N/RClinical: patients who reached weekly average home BP target of SBP ≤135 mmHg and DBP ≤85 mmHg, absolute changes in average weekly home SBP and DBP measurements at the end of the study, absolute changes in pre-, post- and intradialysis BP, absolute changes in IDWG, and adherence to antihypertensive therapyHumanistic: N/R
Quintana-Barcena (2018), Canada [34]	Total: 442; usual care: 138; ProFil: 304	Usual care: 71.2 (12.5); ProFil: 71.9 (12.0)^a^	CKD [eGFR: usual care: 28.2 (10.7); ProFil: 26.8 (9.3)^a^]	A web-based training program for community pharmacists supported by a clinical guide, a discussion forum, the provision of a clinical summary, CKD clinic chart, facilitated access to a pharmacist with expertise in nephrology, the PAIR criteria, access to nephrology pharmacist, community pharmacy medication renewal charts, SCOPE criteria; intervention delivered by community pharmacists as part of a collaborative and multidisciplinary care program	Identifying and assessing DRPs	Economic: N/RClinical: the number and severity of DRPsHumanistic: N/R
Rifkin (2013), USA [48]	Total: 47; usual care: 17; intervention: 30	Usual care: 67.9 ± 8.4; intervention: 68.5 ± 7.5	CKD (eGFR: usual care: 39.4 ± 10.6; intervention: 37.3 ± 14.2)	A novel home-based, Bluetooth-enabled BP monitoring device, laboratory records, VA chart review, VA's EHR (CPRS VISTA); intervention delivered by one pharmacist in collaboration with three physicians	Pharmacist–physician meetings for BP review, patient counselling, medication adjustment, feedback to patient on blood pressure readings	Economic: N/RClinical: SBP, DBP, mean arterial pressure, serum creatinine levels, eGFR, total number of medications, number of blood pressure medications, data exchange and medication adherenceHumanistic: participant feedback on device acceptability
Santschi (2011), Canada [62]	Total: 90; usual care: 42; ProFil: 48	Usual care: 73.3 (±7.7); ProFil: 71.9 (±10.4)	CKD	Three-hour training workshop for community pharmacists, a communication network to facilitate transfer of clinical information between the predialysis clinic and community pharmacists, and a pharmaceutical consultation service; intervention delivered by community pharmacists as part of a collaborative and multidisciplinary care program	Sending a written recommendation to a nephrologist and collaboration between specialized predialysis clinics and community pharmacies	Economic: N/RClinical: SBP, DBP and BP control (<130/80 mmHg), number of antihypertensive drugs and class of antihypertensive medications usedHumanistic: N/R
Sathvik, (2007), India [49]	Total: 102. Number for each group not provided	Usual care: 47.3 ± 17.8; intervention: 50.7 ± 13.7	HD	Written educational materials such as patient information leaflets and take-home medication chart in local language; intervention delivered by a trained clinical pharmacist	Patient counselling and patient education	Economic: N/RClinical: N/RHumanistic: patients’ medication knowledge for name of medications, indication, strength and number of doses to be taken
Skoutakis (1978), USA [38]	Total: 24. Number for each group not provided	Average age was 47 years; does not distinguish between groups	HD	Educational materials (e.g. patient information leaflets) and written reminders; interventions delivered by pharmacists	Patient education, patient counselling on adherence for medication and diet, establishment of patients’ metabolic, dietary and medication profiles, clarification of physicians’ instructions, patient feedback, dose adjustment, and compliance and monitoring for compliance and therapeutic response	Economic: N/RClinical: drug compliance and patients’ biochemical and therapeutic responsesHumanistic: patients’ knowledge and understanding of renal disease, dialysis procedures, and drug and dietary management
Song (2021), South Korea [50]	Total: 100; control: 50; intervention: 50	Control: 54.0 ± 17.4 years; 57.5 (38–67); intervention: 51.0 ± 16.6; 51 (36.3–64)^b^	Mixed (Stage 2–5 and dialysis) [eGFR: control: 9.2 (5.4–20.9); intervention: 8.9 (5.9–20.6)^b^]	Internal guidelines for inpatients, DrugTEAM service model, MEM service, dPCT service, MR service, and educational materials such as written medication guides, timetables, pillboxes and medication diaries; intervention delivered as part of a multidisciplinary intervention	Medication review, communication with healthcare professionals daily, MR, promotion of appropriateness of pharmacotherapy, reduction of medication discrepancies before and after discharge and improve patient compliance and health knowledge, and patient and caregiver were counselled on medication use	Economic: N/RClinical: number of DRPs per patient at discharge, DRP classification, medication adherence for discharge drugs, a composite of acute care utilization (unexpected hospitalization or emergency center visit) within 3 months of discharge, and change in the number of unintentional medication discrepancies at discharge compared with that at the time of admissionHumanistic: N/R
Taber (2021), USA [29]	Total: 136; control: 68; intervention: 68	Control: 51 ± 14; intervention: 50 ± 12	KTR	A smartphone-enabled mobile health app and an accurate list of patients’ medication regimen, which was automatically updated from the EMR, Medicare cost report, South Carolina Medicare CCR for 2019, published by CMS, CPI; intervention delivered by a clinical transplant pharmacist	Clinical pharmacist– led supplemental medication therapy monitoring and management, utilizing a smartphone-enabled mobile health app, integrated with risk-driven televisits and home-based BP and blood glucose monitoring (when applicable), telemonitoring of medications, medical appointment adherence, weekly BP/glucose readings, and scheduling telehealth visits with participants	Economic: charges to payer, multivariable modeling for payer charges and ROIClinical: acute rejections, graft losses, LOS hospitalizationsHumanistic: N/R
Tamilselvan (2021), India [51]	Total: 37; control: 17; test: 20	Control: 48.2 ± 11.3; test: 46.8 ± 12.0	HD	Designed and validated patient information leaflets; intervention delivered by a clinical pharmacist	Patient counselling on CKD, HD, diet and medication	Economic: N/RClinical: N/RHumanistic: HRQoL
Theeranut (2021), Thailand [52]	Total: 334; control: 168; intervention: 166	Control: 68.66 (8.93); intervention: 66.06 (7.94)^a^	CKD [eGFR: control: 53.75 (11.74); intervention: ]	The chronic care model; intervention delivered as part of a collaboration	Pharmacists involved in self-management support and medications	Economic: N/RClinical: mean difference of eGFR from baseline, proportion of patients with eGFR decline greater than 4 mL/min/1.73 m^2^, and difference in CKD stage from baselineHumanistic: N/R
Tuttle (2018), USA [56]	Total: 159; usual care: 75; intervention: 84	Usual care: 69 ± 10; intervention: 70 ± 12	CKD (eGFR: usual care: 42 ± 13; intervention: 40 ± 15)	The chronic care model, an algorithm for the “5As,” process, and the Medication Discrepancy Tool; intervention delivered by a pharmacist	1- to 2-h in-home visit, comprehensive medication review, medication action plan, a personal medication list, patient counselling on medication use, and drug dose evaluation	Economic: N/RClinical: a composite of acute care utilization events (hospitalization or emergency department and urgent care center visits) for 90 days after hospital discharge, individual events comprising the composite, eGFR, serum creatinine levels, UACR, use rates of ACEIs or ARBs and goals for BP, SBP, DBP, HbA1c (diabetic participants), hemoglobin, phosphorus and PTHHumanistic: N/R
van den Oever (2020), Netherlands [57]	Total: 200; control: 100; intervention: 100	Control: 71.2 (21–88); intervention: 66.6 (27–91)^d^	HD	Summary of product characteristics of DA and iron sucrose and anemia treatment guideline; intervention delivered by four pharmacists	Development of treatment algorithms and dose advice	Economic: N/RClinical: median percentage of monthly hemoglobin values in the follow-up period that were in PTR, PSTR, the PTR for iron, PBTR, weekly DA dose, patients with mean dose of ≥90 µg DA per week, iron sucrose dose, all-cause mortality, and number of patients with at least one transfusion during follow-upHumanistic: N/R
Yokum (2008), UK [53]	Total: 34 (SP: 17; intervention = 17)	SP: 47.6 ± 14.4; intervention: 51.1 ± 12.7	HD	Medication card and a phosphate management protocol designed by the multidisciplinary renal research team; intervention delivered in collaboration with a renal consultant and a dietitian	Medication adjustment and patient counselling for meals and medications	Economic: N/RClinical: serum levels of phosphate, calcium-phosphate product, corrected calcium, intact PTH; medication adjustmentsHumanistic: N/R

Unless otherwise stated, data presented as mean ± standard deviation.

^a^Data presented as mean (standard deviation); ^b^data presented as median (IQR); ^c^data presented as median (5th–95th percentiles); ^d^data presented as median and range; ^e^data presented as mean (range).

%CV: coefficient of variation; 5As: assessment, advice, agreement, assistance and arrangements; ACEI: angiotensin-converting enzyme inhibitor; ARB: angiotensin-receptor blocker; BP: blood pressure; CCR: cost-to-charge ratio; CMS: Centres for Medicare & Medicaid Services; CPI: consumer price index; CRP: C-reactive protein; CV: cardiovascular; DA: darbepoetin alfa; dPCT: discharge pharmaceutical care transition; DRAP: drug renal alert pharmacy; DRPs: drug-related problems; drugTEAM: drug therapy evaluation and management; DUE: drug use evaluation; eCDSS: electronic clinical decision support system; EHR: electronic health record; EMR: electronic medical record; ESKD: end-stage kidney disease; HbA1C: glycated hemoglobin; HD: hemodialysis; ICER: Incremental Cost-Effectiveness Ratio; IDW: interdialytic weight gain; iPTH: intact parathyroid hormone; IQR: interquartile range; IST: immunosuppressant; JAMA: *Journal of the American Medical Association*; KDIGO: Kidney Disease: Improving Global Outcomes; KDOQI: Kidney Disease Outcomes Quality Initiative; KTR: kidney transplant recipient; LOS: length of stay; MCG: Medical College of Georgia; MEM: medication evaluation and management; MR: medication reconciliation; MTM: medication therapy management; N/R: not reported; PAIR: pharmacotherapy assessment in chronic renal disease; PBD: pharmacy-based dosing; PBTR: percentage below target range; PCP: primary care provider; PIMS: pharmacy information management system; PSTR: percentage in supratherapeutic range; PTH: parathyroid hormone; PTR: percentage within target range; QALY: quality adjusted life-year; ROI: return on investment; SCOPE: Severity Categorization for Pharmaceutical Evaluation; SP: standard practice; TAC: tacrolimus; TSAT: transferrin saturation; UACR: urinary albumin-to-creatinine ratio; VA: Veterans’ Administration; WHO-FIP: World Health Organization-International Pharmaceutical Federation.

### Overview of outcome domains

A plethora of outcomes were identified: 10 economic, 211 clinical and 18 humanistic, which are summarized in Supplementary data, Table S5. The clinical outcomes were grouped through discussion into 39 individual clinical outcome domains (Table 2) and are listed in Supplementary data, Table S6.

**Table 2:** Direction of effect plot for clinical outcomes domains.

Clinical outcome domain	Number of studies	Direction of effect	Summary of findings
Adverse events	2	→	Four outcomes with no effect
Appropriate medical treatment	1	→	Mixed findings—positive effect direction for two outcomes and no effect for two outcomes
Blood pressure	8	→	Mixed findings—two outcomes with positive effect direction and 15 had no effect; findings for SBP (Fig. 2A and Supplementary Fig. S2A) and DBP (Figs. 2B and Supplementary Fig. S2B) were meta-analyzed and not included in the direction effect plot^a^
Clinical blood markers	7	→	Mixed findings—11 outcomes with positive effect direction and 21 had no effect; findings for LDL cholesterol (Fig. 3A) and hemoglobin (Fig. 3D and Supplementary Fig. S3B) were meta-analyzed and not included in the direction effect plot
Clinical grading of pallor	1	↑	One outcome with positive effect direction
Comorbidity management	1	→	One outcome with no effect
Compliance pattern	1	↑	One outcome with positive effect direction
Composite of adverse events	3	→	Three outcomes with no effect
Cardiovascular risk	1	→	Mixed findings—one outcome with positive effect direction and one had no effect
Data exchange	1	↑	One outcome reported positive effect direction
Dialysis adherence	1	↑	Five outcomes with a positive effect direction and one had no effect
Dose adjustment	1	→	Mixed findings—one outcome with positive effect direction and two outcomes with no effect
Drug-related problems	3	→	Mixed findings—two outcomes with positive effect direction and two outcomes with no effect
Graft function	2	→	Mixed findings—two outcomes with positive effect direction and two outcomes with no effect
Healthcare utilization	4	→	Seven outcomes which demonstrated no effect
Hospitalization	8	→	Mixed findings—four outcomes with positive effect direction and five had no effect
Interdialytic weight	2	→	Mixed findings—one outcome with positive effect direction and one had no effect
Immunosuppressant concentration	2	→	Mixed findings—one outcome with positive effect direction and two had no effect
Incorrect CKD staging	1	→	No effect reported for one outcome
Infection	2	→	No effect reported for three outcomes
Kidney function	3	→	Mixed findings—one outcome with positive effect direction and two outcomes with no effect; findings for eGFR (Fig. 3B) and for creatinine (Fig. 3C and Supplementary Fig. S3A) were meta-analyzed and not included in the direction of effect plot^a^
Kidney progression	4	→	Mixed findings—two outcomes with positive effect direction and three had no effect
Lipid screening	1	→	No effect reported for one outcome
Medication adherence	11	→	Mixed findings—five outcomes with positive effect direction and seven had no effect
Medication changes	2	↑	Mixed findings—three outcomes with positive effect direction and one outcome with no effect
Medication class	1	→	No effect reported for one outcome
Medication discontinuation	1	→	No effect reported for one outcome
Medication discrepancy	1	↑	One outcome reported positive effect direction
Medication dose	1	→	Mixed findings—two outcomes with positive effect direction and one outcome with no effect
Medication error	3	↑	Five outcomes reported positive effect direction
Medication initiation	3	→	Mixed findings—one outcome with positive effect direction and five had no effect
Medication use	3	→	No effect reported for six outcomes
Mortality	5	→	No effect reported for five outcomes
Number of medications	5	→	Mixed findings—three outcomes with positive effect direction and three outcomes with no effect
Number of patients with at least one transfusion during follow-up	1	↑	One outcome reported positive effect direction
Patients’ biochemical and therapeutic responses	1	↑	One outcome reported positive effect direction
Proteinuria screening	1	→	No effect reported for one outcome
Smoking cessation	1	↑	One outcome with positive effect direction
Tacrolimus intrapatient variability	2	→	Mixed findings—two outcomes with positive effect direction and three outcomes with no effect

To assess for the overall direction of effect, an upward arrow (↑) signified a positive health effect; a downward arrow (↓) indicated a negative health effect; and a sideways arrow (→) signified there was no clear effect/conflicting findings [22]. If the direction of effect for multiple outcomes across studies for an umbrella outcome was the same, then that effect direction was reported [22]. However, if there was variation across studies, then the direction of effect was reported if the majority (≥70%) reported a similar direction; a sideways arrow was used (→) if <70% of outcomes reported a constant direction of effect [22].

a Creatinine, DBP and SBP for Okoro *et al.* 2022 at 6 months were only included, as data from this timepoint were not meta-analyzed.

### Quality assessment

The quality assessment for the included studies varied (Supplementary data, Table S7). The majority (20/32, 63%) of studies were of poor quality [16, 30, 35, 37–53], five (16%) studies were fair quality [17, 54–57] and seven (22%) studies were rated good [27, 33, 58–62]. Most studies (20/32, 63%) had a high risk of bias due to no intention-to-treat (ITT) analysis being applied.

### Clinical outcomes

#### Narrative synthesis

Table 2 shows the effect direction plot for various clinical outcomes domains. Outcome domains that were reported in more than four studies are narratively summarized in Table 3.

**Table 3:** Narrative synthesis of clinical outcomes.

Outcomes	Findings
Blood pressure	Eight RCTs reported different outcomes for blood pressure [17, 41, 47, 48, 56, 60–62], with two (25%) studies reporting a favorable effect on blood pressure [17, 47]
	- Qudah *et al.* reported there was no difference between intervention and control groups for absolute changes in pre-dialysis, post-dialysis and intradialysis DBP and SBP [47]. However, more patients reached weekly average home blood pressure target of SBP ≤135 and DBP ≤85 mmHg in the intervention group (46.7%) vs those in the control group (14.3%), (*P* = .02) at the end of trial, with an odds ratio of 5.14 (95% CI 1.2–21.8) [47]
	- Rifkin *et al.* reported no difference between intervention and control groups for mean arterial pressure [48]
	- Pharmacist interventions did not lead to more participants achieving the desired blood pressure goal, in comparison with the control group [17, 41, 56, 60–62]
	- Okoro *et al.* demonstrated that the group that received pharmaceutical care had no improvement in DBP at 6 months, although a reduction SBP was reported at 6 months, relative to the control group (–8.5 mmHg, *P* = .029) [17]
	- No difference was reported by Cooney *et al.* between intervention and control groups for SBP among participants with a baseline pressure >130/80 mmHg [60]
Clinical blood markers	Mixed findings were reported for clinical blood markers
	- Alshogran *et al.* reported no differences between the pharmacist intervention group and the usual care group for albumin, calcium, calcium*phosphate product, corrected calcium, parathyroid hormone (PTH), potassium and sodium levels [16]. However, there was a reduction in phosphorous (intervention: 4.5 ± 1.3 mg/dL vs control: 5.2 ± 1.6 mg/dL; *P* = .02) and urea levels (intervention: 112.2 ± 46.5 mg/dL vs control: 144.8 ± 45.6 mg/dL; *P* = .001) [16]
	- Yokum *et al.* reported no difference between intervention and control groups for corrected calcium levels, and the intervention group involving the pharmacist led to a reduction in phosphate levels, relative to the usual care group (–0.22 ± 0.67 mmol/L vs 0.19 ± 0.32 mmol/L, *P* = .03) [53]. Moreover, they found a reduction in calcium*phosphate product levels in the intervention group, compared with the usual care group (–0.58 ± 1.62 vs 0.41 ± 0.81, *P* = .04), although there was no difference between them for intact PTH levels [53]
	- Tuttle *et al.* reported there were no differences between intervention and usual care groups for attaining goals for hemoglobin, HbA1C (for diabetic participants), phosphorus and PTH [56]
	- In contrast, Al Hamarneh *et al.* found that the between group difference of intervention and control groups for HbA1C was 0.7 (0.4–0.9) (95% CI) % (*P* < .001) [58], whilst Lalonde *et al.* reported no difference between intervention and control groups [33]
	- Cooney *et al.* reported a difference between the number of participants who had their phosphorous measured during the study period (control group: 527/1129 vs intervention group: 680/1070, *P* < .001) and who had their PTH measured during the trial (control group: 182/1129 vs intervention group: 502/1070, *P* < .001) [60]
	- Armstrong *et al.* reported no difference between treatment and control groups for hematocrit, serum ferritin, and serum transferrin saturation (TSAT) [39]
	- Similarly, Marouf *et al.* found no differences for serum ferritin between intervention and control group, as well as for serum folate and vitamin B12 levels, although pharmacist intervention did result in an increase in TSAT (intervention group: 47 ± 0.17% vs control group: 40 ± 0.13%, *P* < .05) [46]
	- van den Oever *et al.* reported that pharmacist intervention resulted in an increase in the percentage in therapeutic range per patient for hemoglobin [intervention group: 38.5% (16.7–53.9) vs control group: 23.1% (9.1–46.2), *P* = .001] and for the percentage range per patient for iron [intervention group: 21.1% (7.7–38.9) vs control group: 8.3% (0.0–30.8), *P* = .003], whilst a lower percentage in supra-therapeutic range per patient for hemoglobin was reported for the those that received an intervention from the pharmacist [intervention group: 0.0% (0.0–12.9) vs control group: 7.7% (0.0–27.3), *P* = .034] [57]. There was no difference between the groups for the percentage below the target range for hemoglobin per person; data expressed by van den Oever *et al.* was median (interquartile range) [57]
Hospitalization	Three studies reported on duration of hospitalization
	- Pai *et al.* and Taber *et al.* found there was no differences between intervention and control groups [29, 36], although Chisholm-Burns *et al.* identified that the proportion of patients in the control group who spent at least one day in hospital during the study was significantly greater than those in the intervention group (57.3% vs 23.9%, respectively; x^2^ = 15.66; df = 1, *P* < .001) [59]
	- Two RCTs demonstrated that pharmacist interventions led to a reduction in hospitalization rate [28, 36], with Gonzales *et al.* reporting lower rates of hospitalization [1.08 (control) vs 0.65 (intervention) hospitalizations per patient-year, *P* = .007] [28], and Pai *et al.* found there were fewer all-cause hospitalizations for patients who received pharmaceutical care, relative to standard care (1.8 ± 2.4 vs 3.1 ± 3 hospitalizations, *P* = .02), during a 2-year study [36]. Two studies reported no differences between intervention and usual care groups for the number of hospitalizations [55, 56]
	- Alshogran *et al.* found that pharmacist interventions resulted in a lower mean number of hospital and room admissions, in comparison with the control group (0.54 ± 0.07 vs 0.78 ± 0.26, *P* < .001) [16]
	- Bessa *et al.* reported there was no difference between the pharmaceutical care and control groups for hospital readmissions [40]
Medication adherence	Mixed findings were reported for medication adherence in 11 studies [16, 17, 60, 31, 37, 38, 40, 47, 48, 50, 59]. No statistically significant difference between intervention and control groups was reported in six studies [17, 40, 47, 48, 50, 60]. Favorable effects on medication adherence were reported in five studies
	- Chisholm-Burns *et al.* demonstrated that renal transplant recipients who received an intervention from the pharmacist had a greater adherence to immunosuppressant therapy, relative to the control group, throughout the 1-year study period (t = 2.73; df = 133; *P* = .0071) [59]
	- Alshogran *et al.* reported a difference in medication adherence at follow-up in the pharmacist intervention group (194.7 ± 15.5) vs the control group (121.1 ± 61.2) (*P* < .001) [16]
	- In a multicenter RCT, Mateti *et al.* found that the pharmaceutical care group had a increase in medication adherence rates at repeated time intervals, relative to the usual care group, at academic (*P* < .001), government (*P* = .022) and corporate (*P* < .001) hospitals [31]
	- Skoutakis *et al.* demonstrated that mean drug compliance during Phase II for hemodialysis patients who received interventions from the pharmacist was higher than those who received usual care during Phase I [84 (4.04)% vs 61 (5.08)%, *P* < .001], respectively [38]. Moreover, in Phase III, Group I participants who continued to receive pharmaceutical care from the pharmacist had a greater mean compliance than those in Group II that reverted to usual care [90 (2.01)% vs 74 (3.03)%, *P* < .001], respectively [38]
	- Chisholm *et al.* reported that following 1-year post-transplantation, patients that received interventions from the clinical pharmacist had a higher mean compliance rate for their immunosuppressant medications than those who did not receive pharmacist input (96.1 ± 4.7% vs 81.6 ± 11.5%, *P* < .001) [37]
Mortality	Five studies reported that there were no differences between the pharmacist and usual care groups for mortality [31, 40, 55, 57, 60].
Number of medications	Pharmacist interventions yielded mixed results for the number of medications
	- Santschi *et al.* found that the number of antihypertensives was similar in both groups at the end of the trial [62]
	- Rifkin *et al.* reported similar findings, with there being no difference in the number of antihypertensives and total number of medications [48]
	- In contrast, Pai *et al.* demonstrated that intervention in the pharmaceutical group was associated with a reduction of 14% fewer concurrent medications, relative to the usual care group (*P* < .05) [36]
	- Cooney *et al.* reported that pharmacist intervention group resulted in more patients with poorly controlled hypertension prescribed antihypertensives, relative to those in the control group (*P* = .02) [60]
	- Cypes *et al.* demonstrated more medicines required pharmacist intervention, than the control group (*P*-value not reported) [44]

#### Meta-analysis

A meta-analysis was appropriate for the following: blood pressure, low-density lipoprotein (LDL) cholesterol, estimated glomerular filtration rate (eGFR), creatinine and hemoglobin.

#### Blood pressure

**Figure 2:**
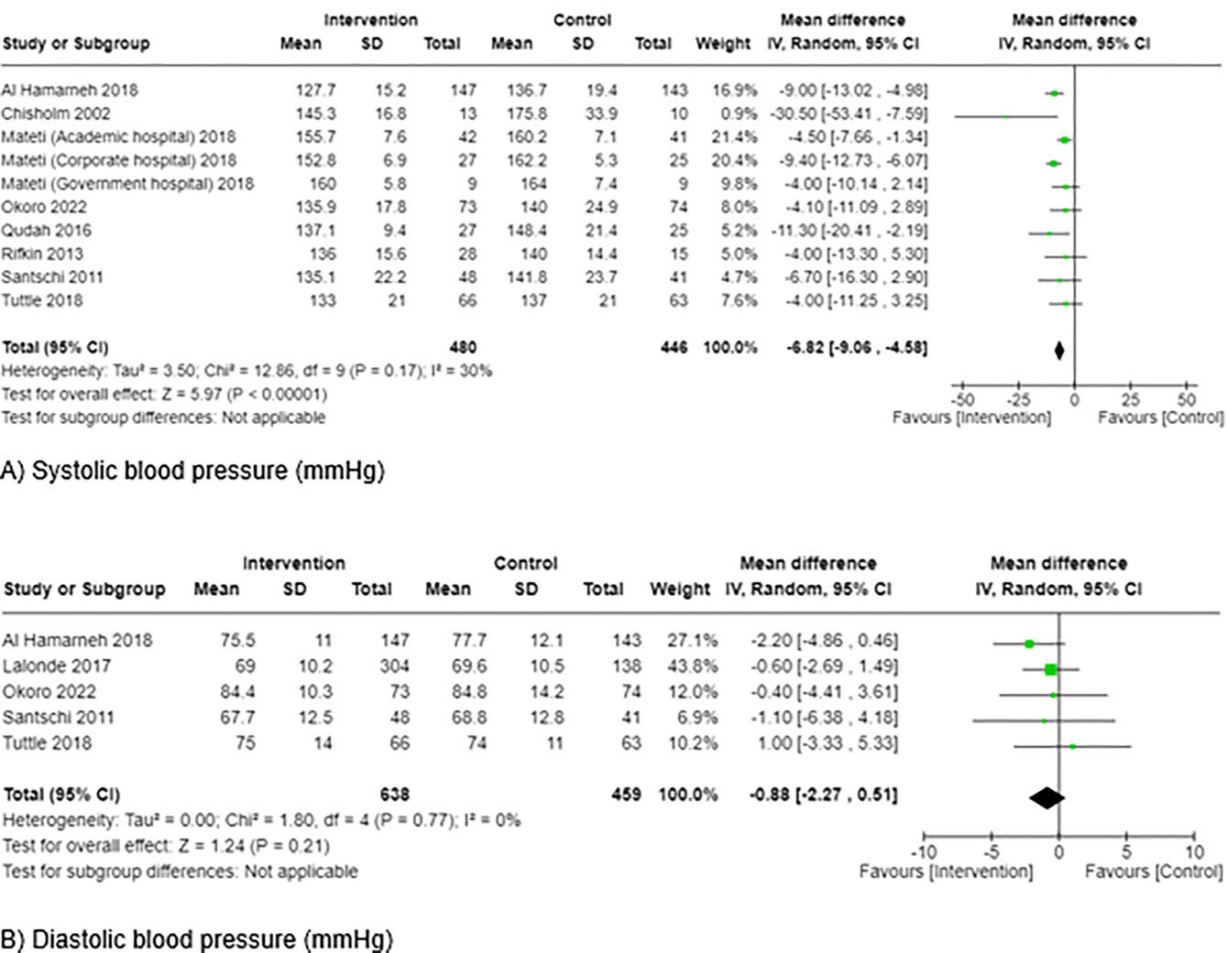
Forest plots for (**A**) systolic blood pressure after removal of Lalonde *et al*. 2017 and for (**B**) diastolic blood pressure following removal of four reports based on two studies. (A) Lalonde *et al*. was removed because it resulted in the largest reduction in the *I*^2^ value and went below 40% [25]. (B) Four reports based on two studies were removed because the “one-study-removed” approach for each study did not cause the *I*^2^ value to fall below 40% [25]; studies with a “poor” quality rating were subsequently removed [26].

Nine studies, with a total of 1368 participants, reported systolic blood pressure (SBP) (–6.06 mmHg, 95% CI –8.45, –3.67, *P* < .00001, *I*^2^ = 48%; Supplementary data, Fig. S2A) [17, 31, 33, 42, 47, 48, 56, 58, 62], however, moderate heterogeneity was identified. A “one-study removed” sensitivity analysis meant Lalonde *et al.* was removed [33]. This resulted in eight studies with 926 participants being meta-analyzed (Fig. 2A) [17, 31, 42, 47, 48, 56, 58, 62]. The overall pooled estimate demonstrated a statistically significant difference in SBP between the groups, favoring pharmacist interventions (–6.82 mmHg, 95% CI –9.06, –4.58, *P* < .00001, *I*^2^ = 30%).

Nine RCTs, including a total of 1368 participants, reported diastolic blood pressure (DBP) [17, 31, 33, 42, 47, 48, 56, 58, 62]. Supplementary data, Fig. S2B illustrates the overall pooled estimate (–3.14 mmHg, 95% CI –5.03, –1.23, *P* = .001, *I*^2^ = 65%). Sensitivity analysis was conducted, where the “one-study removed” procedure for each trial found that heterogeneity was >40%. Further analysis was performed to reduce the heterogeneity to ≤40% and four reports based on two studies were removed (Fig. 2B). In the five remaining studies, including 1097 participants, the intervention effect on DBP was non-significant (–0.88 mmHg, 95% CI –2.27, 0.51, *P* = .21, *I*^2^ = 0%).

#### Other clinical outcomes

**Figure 3:**
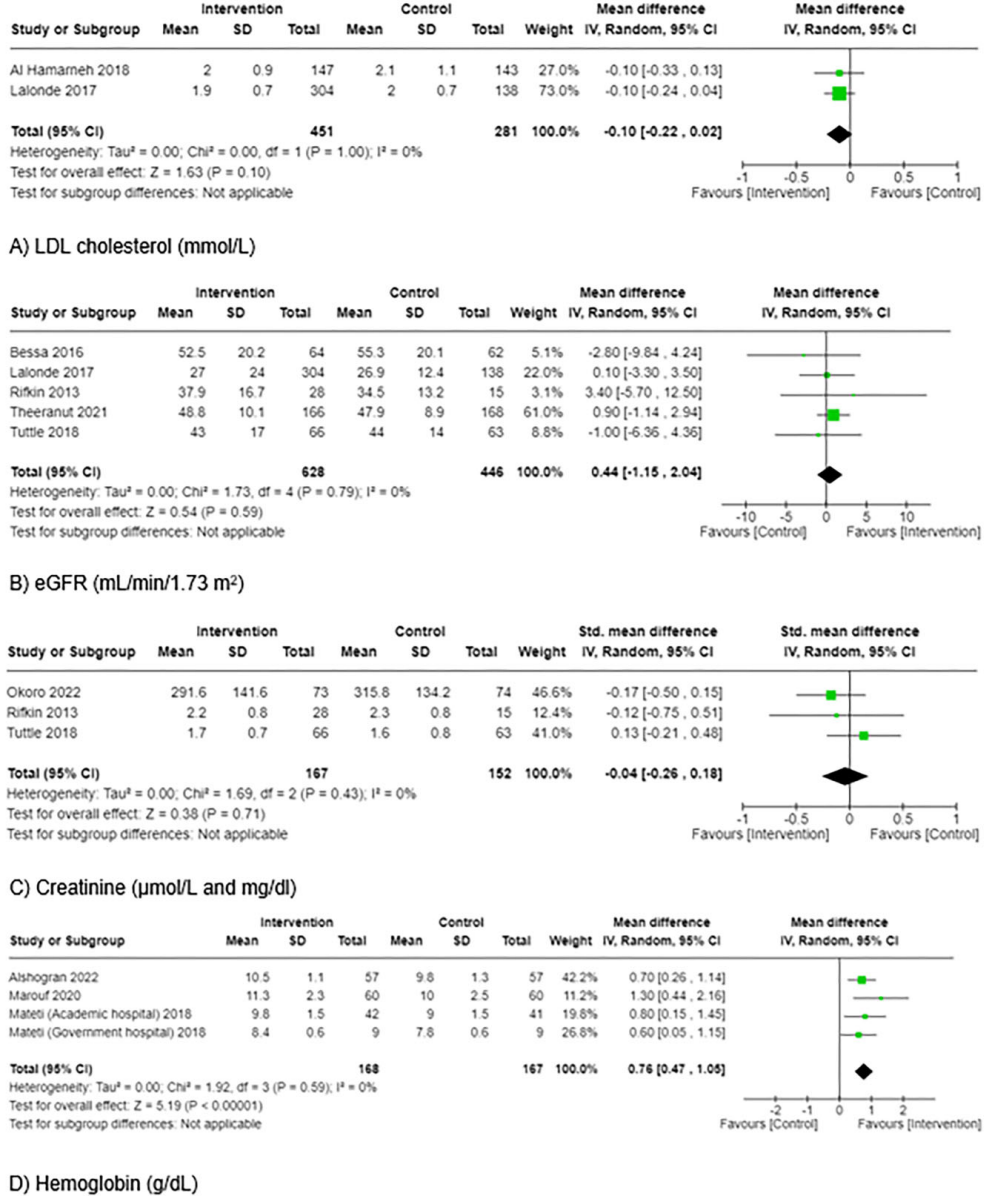
Forest plots for other clinical outcomes: (**A**) LDL cholesterol, (**B**) eGFR, (**C**) creatinine and (**D**) hemoglobin. In (C) Alshogran *et al.* 2022 was removed because it resulted in the largest decrease in the *I*^2^ value and went below 40% [25]; in (D) Mateti *et al.* (corporate hospital) 2018 was removed because it went below the threshold of 40% [25].

Two studies, including a total of 732 participants, assessed the impact on LDL cholesterol (Fig. 3A) [33, 58]. No statistically significant difference between the intervention or control groups (–0.10 mmol/L, 95% CI –0.22, 0.02, *P* = .10, *I*^2^ = 0%) was found.

Kidney function was reported as eGFR and creatinine. Five trials, including a total of 1074 participants, reported eGFR [33, 40, 48, 52, 56]. Although the point estimate of three studies indicated an increase in eGFR in the pharmacist intervention groups [33, 48, 52], the pooled analyses found no significant effect (0.44 mL/min/1.73 m^2^, 95% CI –1.15, 2.04, *P* = .59, *I*^2^ = 0%) (Fig. 3B). Four trials, including a total of 433 participants, reported creatinine (Supplementary data, Fig. S3A) [16, 17, 48, 56]. After performing the “one-study removed” procedure, the overall point estimate from three studies, including a total of 319 participants (Fig. 3C) [17, 48, 56] indicated no difference between intervention and control groups (–0.04 mg/dL, 95% CI –0.26, 0.18, *P* = .71, *I*^2^ = 0%).

Three studies, including a total of 387 participants, reported hemoglobin (Supplementary data, Fig. S3B) [16, 31, 46]. There was a significant between group difference, although heterogeneity was evident (I^2^ = 42%). After conducting the “one-study removed” approach, Fig. 3D shows that the pooled effect of pharmacist interventions led to a statistically significant improvement in hemoglobin levels, relative to the control groups (0.76 g/dL, 95% CI 0.47, 1.05, *P* < .00001, *I*^2^ = 0%) in three studies, including a total of 335 participants.

### Economic and humanistic outcomes

#### Narrative synthesis

Economic outcomes were reported only in four studies that involved pharmacist interventions [29, 32, 36, 59] and are summarized in Table 4. Mixed findings were reported, with Taber *et al.* demonstrating that pharmacist interventions resulted in a return of investment (ROI) of ∼$4 for every $1 spent for the intervention and lower hospitalization costs of $390 489 for the payer, relative to the estimated hospitalization costs of $870 468 in the control group [29]. However, Pai *et al*. reported that pharmaceutical care provided by a clinical pharmacist did not result in a statistically significant reduction in the mean cost of concomitant drugs [36]. Table 5 provides a summary of the findings for humanistic outcomes, which also yielded mixed results. Alshogran *et al.* found that pharmacist interventions led to an improvement in disease awareness and adherence behaviors in hemodialysis patients [16]. In contrast, for HRQoL, four studies reported an improvement in HRQoL [16, 30, 45, 51], whilst two studies reported no difference between groups [35, 60]; one study did not report the total HRQoL score, but reported an improvement in certain domains [30].

**Table 4:** Narrative synthesis of economic outcomes.

Outcomes	Findings
Cost of all concomitant drugs	Pai *et al.* demonstrated that pharmaceutical care reduced the mean cost of concomitant drugs by approximately $6 in comparison with the standard care group, although this difference was not significant [36]
Cost-saving	Chisholm-Burns *et al.* reported in a cost-savings projection sub-analysis that the total costs associated with hospitalization would have been higher in the control group by an estimated $28 000 per month, relative to the group that received interventions delivered by a clinical pharmacist [59]
Cost data, cost-effectiveness grid and plane, ICER, QALY and utility values	A multicenter RCT conducted in hemodialysis centers at academic, government and corporate hospitals in India found there were no statistically significant differences for one year utility values and quality-adjusted life year (QALY) between the intervention and usual care groups [32]. However, the incremental cost-effectiveness ratio (ICER) and the cost-effectiveness grid and plane indicated that pharmaceutical care was more effective, but costlier [32]. Mateti *et al.* also found that the annual cost of medications (usual care: median, interquartile range, 19 790 (5574.25) Indian rupees (INR) vs pharmaceutical care: 24 623 (8820.75) INR, *P* = .033) and laboratory investigations [usual care: 2552.50 (806.50) INR vs 2691.50 (727) INR, *P* = .009] were more expensive in the pharmaceutical care group than the usual care group in the academic hospital, but there were no statistically significant differences between the groups for the corporate and government hospitals [32]
Charges to payer, multivariable modelling for payer charges, ROI	Taber *et al.* reported several findings, which include lower hospitalization costs of $390 489 in the intervention arm for the payer vs estimated hospitalization costs of $870 468 in the control arm; a 49% lower charge risk in the adjusted multivariable model intervention arm when compared with the control arm (relative risk 0.51, 95% CI 0.28–0.91; *P* = .022); and a ROI of $4.30 for every $1 spent for the intervention [29]

**Table 5:** Narrative synthesis of humanistic outcomes.

Outcomes	Findings
HRQoL	HRQoL was reported in six studies [16, 30, 35, 45, 51, 60], four of which demonstrated that pharmacist interventions led to a favorable improvement in HRQoL [16, 30, 45, 51]. However, one study did not report the total HRQoL score, but reported an improvement in certain domains [30]. Two studies reported no difference between groups for HRQoL [35, 60]
Patient knowledge	Sathvik *et al.* found that pharmacist interventions furthered medication knowledge in patients [49]. Similar findings were also reported by Skoutakis *et al.*, where there was an improvement in patients’ knowledge and understanding of kidney disease, dialysis procedures, and drug and dietary management [38]
Patient satisfaction	Three studies reported satisfaction with care provided by pharmacists: Cooney *et al.* identified that 92% of participants felt the information provided to them was useful [60]; Okoro *et al.* found interventions delivered by pharmacists led to an improvement in patients’ satisfaction (*P* < .001) [17]; and one of the findings from Chang *et al.* indicated that patients were highly satisfied with the care they received from the pharmacist [41]. Two studies also illustrated how the use of technology as part of the interventions delivered by pharmacist led to high participant satisfaction [27, 48]
Adherence behaviors and disease awareness	Alshogran *et al*. demonstrated that interventions delivered by clinical pharmacists helped improve the perception of adherence behaviors among hemodialysis patients for diet, fluid intake, dialysis management and taking medicines as prescribed, relative to the control group, (*P* < .001 for all), as well as raising awareness of end-stage kidney disease [16]

## DISCUSSION

### Summary of key findings

Since the last systematic review in this area was published in 2019 [13], findings from 11 new RCTs have been published [16, 17, 27, 43, 44, 46, 50–52, 57, 61]. This systematic review and meta-analysis included 32 trials and yielded several novel findings. Firstly, the interventions delivered by pharmacists were wide ranging, and encompassed patient counselling, development of treatment algorithms, dose adjustments, patient education, medication reviews, medicine optimization and the management of comorbidities. The review identified a vast number of outcomes including 10 economic, 211 clinical and 18 humanistic outcomes. The heterogeneity and plethora of clinical outcomes limited interpretation and pooling of the majority of the data and meant that a direction of effect plot was used to summarize the overall direction of effect for several clinical outcome domains. No effect was reported for outcomes such as infection and mortality, whilst an overall positive effect was reported for several outcome domains, including smoking cessation and for medication initiation. Mixed findings were reported for economic outcomes, although pharmacist interventions led to an improvement in humanistic outcomes such as patient satisfaction and patient knowledge. The meta-analyses demonstrated that pharmacist interventions resulted in significant improvements in SBP and hemoglobin levels. However, no effects were reported for DBP, eGFR, creatinine and LDL cholesterol levels. Overall, pharmacist interventions yielded mixed results for outcomes, although interpretation is limited by low study quality and high heterogeneity across studies.

### Interpretation of key findings

Interventions delivered by pharmacists led to a significant and clinically relevant reduction of SBP in the region of ∼6 mmHg. Reducing blood pressure and improving hypertension outcomes in CKD is important, as hypertension is a risk factor for CKD and increases the risk of progression [63]. The mechanisms for how pharmacist-led interventions impact blood pressure are likely multifaceted, although likely are driven by better medicine management. Chisholm *et al.* illustrated that obtaining medication histories, providing medication recommendations to nephrologists and counselling patients to ensure they adhere to their medication resulted in reduction of SBP [42]. Qudah *et al.* illustrated in hemodialysis patients that collaboration between the pharmacist and physician to optimize antihypertensive therapy, patient education and patient counselling to ensure adherence to antihypertensives, and fluid and salt restrictions led to a reduction of SBP [47].

Anemia is common in people with CKD [64], so it is favorable that results showed pharmacist interventions led to improved hemoglobin levels. Marouf *et al.* demonstrated that an increase could have been attributed to dose adjustments to erythropoietin and iron, counselling/education to enhance medication adherence or regular monitoring [46]. Similarly, Mateti *et al.* attributed an improvement in hemoglobin levels due to medication adherence and frequent monitoring of anemia [31].

Another frequently measured outcome was medication adherence. Findings showed a mixed effect of pharmacist interventions. Okoro *et al.* found that pharmacist interventions did not lead to an improvement in antihypertensive medication adherence at 12 months, potentially suggesting that interventions delivered may require more intensive individualized follow-ups [17]. In contrast, Chisholm-Burns *et al.* demonstrated an improvement in medication adherence in those that received a 1-year behavioral contract intervention delivered by a clinical pharmacist [59].

Pharmacist interventions had no favorable effect on kidney function. This could be attributed to only three (out of five) studies reporting the use of angiotensin-converting enzyme inhibitors or angiotensin-receptor blockers in patients [17, 48, 56], which reduce proteinuria and subsequently slow the progression of CKD [65]. Newer pharmacological therapies such as SGLT2Is were not used in any studies in the review, which may slow CKD progression [66, 67]. It is likely that future meta-analyses involving these newer pharmacological therapies may find favorable effects on kidney function.

There were several weaknesses with the included studies. Many had a high risk of bias and were of poor quality for predominantly not incorporating ITT analysis [21]. ITT analysis ensures that groups are analyzed exactly as they were randomized [68], and ensures a prognostic balance and sample size are maintained [69]. Future studies should ensure that ITT analysis is performed as per recommended guidance. Interestingly, none of the studies involved deprescribing as a specific element of the intervention. Multimorbidity or multiple long-term conditions are common in people with CKD [70] and increases the risk of polypharmacy [71], where the latter is associated with increased side effects, adverse drug reactions, reduced medication adherence and drug interactions [72]. Deprescribing describes the systematic process of identifying and stopping or tapering medications in instances in which the possible or existing harms outweigh the possible or existing benefits, with the aim of improving of patient outcomes and reducing the burden of polypharmacy [73], and has been shown to be effective in other conditions [74, 75]. The failure to de-intensify therapy when appropriate can result in poor patient outcomes [76]. Future RCTs could investigate deprescribing as an intervention in people with CKD.

### Strengths and limitations

This review has several strengths. A comprehensive search strategy was devised, multiple databases were used, and a broad range of outcomes based on the ECHO model were used to provide a comprehensive overview of pharmacist interventions. We included participants across all stages of CKD and only RCTs were included, which ensure the highest level of evidence for clinical trial design and a reduced risk for systematic errors [77]. This review encompassed RCTs that were conducted in 13 countries across five continents, demonstrating internationally how pharmacist interventions can impact CKD patients. The structures and processes in the studies were described in detail, providing context and information of the various resources and interventions delivered by pharmacists. The review was prospectively registered and a detailed protocol [18] adhered to the PRISMA‐P standards [78]. The current systematic review was conducted per the PRISMA 2020 statement [19].

This review has several limitations. Specific databases (e.g. OpenGrey) containing grey literature were not searched, although we did include Google Scholar, and studies that were not in English were excluded, thereby potentially limiting the inclusion of studies in other languages. Furthermore, only RCTs in which CKD was the primary condition were included. Consequently, studies which had participants with CKD as a comorbidity in a multimorbid context were omitted as it would not be feasible to determine the impact of pharmacist interventions explicitly on CKD patients and this may have led to a sampling error. Only one RCT was conducted in the UK [53], which could limit the applicability of the review findings to the UK healthcare system. The interventions (and processes) delivered by pharmacists were multifaceted, which made it difficult to ascertain the impact of a particular intervention on certain outcomes. Whilst the purpose of this review was to look at the effects of pharmacist interventions in CKD, the use of different types of medications (e.g. licensed in different countries) likely had different efficacies on outcomes irrespective of the pharmacist involvement, which we were unable to capture in this work. Only one study addressed discrepancies between pharmacists and other healthcare professionals and how they were resolved [47].

### Implications for clinical practice

This review has several implications for clinical practice. The review demonstrated the interventions pharmacists provide may improve economic (e.g. hospitalization costs), clinical (e.g. SBP) and humanistic outcomes (e.g. patient satisfaction). As part of future healthcare planning (e.g. the NHS Long Term Plan), more pharmacists are likely to become incorporated in multidisciplinary teams in primary care networks, hospital pharmacists will extend their practice into primary care and community pharmacists will continue to provide high quality care for minor ailments [12]. Moreover, pharmacist prescribers will increasingly become an integral component of multidisciplinary teams across primary care networks [12]. The increased utilization of pharmacists will help lessen the burden on the healthcare systems, and in the UK, from 2026, newly registered pharmacists will become independent prescribers [79], thus helping ease pressure on the healthcare system. As such, although this review identified three studies that reported pharmacist prescribing as an intervention [41, 58, 60], this is likely to be more common in the upcoming decade. Interestingly, conflict in decision-making between pharmacists and clinicians was poorly reported across studies; this is an important element of effective healthcare management and further work should explore this. The findings described here strengthen the evidence and importance of pharmacists in CKD management.

Whilst none of the RCTs in this review explored the impact of pharmacist deprescribing in CKD, several studies have looked at its effect. A deprescription tool formulated by a nephrologist and a clinical pharmacist, which was used for a deprescription program for hemodialysis patients, demonstrated there was a statistically significant reduction in the number of medications, the number of pills and pill burden, which was assessed using the Living Medication Questionnaire-Visual Analogue Scale score [80]. A study by McIntyre *et al.* found that a deprescribing tool developed by the nephrology team for hemodialysis patients, which included two pharmacists, resulted in the deprescription of 31 of 40 (78%) target medications after 4 weeks in 35 patients [81].

### Implications for research

This systematic review has several implications for research. Significant heterogeneity was observed for outcome measures limiting synthesis and interpretation of data. For example, in 11 studies where medication adherence was assessed [16, 17, 31, 37, 38, 40, 47, 48, 50, 59, 60], five separate measures were used: variations of the Morisky Medication Scale [17, 31, 47, 48, 50, 60], pharmacy refill records [37, 59], Basel Assessment of Adherence to Immunosuppressive Medication Scale [40], a comparison of the quantity of medications prescribed with those present in containers [38] and the end-stage renal disease adherence questionnaire [16]. Moreover, three separate outcome measures were used to assess HRQoL (e.g. the Medical Outcome Study 36-Item Short-Form Health Survey) [45]. The plethora of outcome measures used suggests that better standardization [e.g. through the use of a core outcome set (COS) [82]] is required to facilitate the consolidation of pharmacy research. Nephrology is well adept to the use of COS and the Standardized Outcomes in Nephrology initiative was launched to improve consistency in the reporting of outcomes that are essential to healthcare professionals and people with CKD for trials [83]. However, no pharmacist-specific COS have yet to be developed for use in CKD. Complex, future (potentially adaptive-based platform) RCTs are needed to determine what unique aspects of interventions delivered by pharmacists are effective. Alternatively, a realist evaluation/review [84] of interventions could be undertaken to better inform the design of future interventions.

## CONCLUSION

Findings showed pharmacist interventions had mixed results for various outcomes, with a statistically significant improvement in SBP and hemoglobin levels, but not in DBP, LDL cholesterol and creatinine levels. Mixed results were reported for other clinical, economic and humanistic outcomes. Future studies should be more robustly designed and take into consideration the role of the pharmacist in prescribing and deprescribing, the findings of which will help inform research and clinical practice.

## Supplementary Material

gfae221_Supplemental_File

## Data Availability

Full extracted data from included studies can be requested from the authors.
