# Supplementary material for: Effect of pharmacist interventions in chronic kidney disease: a meta-analysis
Source: Nephrol Dial Transplant. 2024 Oct 9;40(5):884–907. doi: 10.1093/ndt/gfae221 (PMC12209859; doi:10.1093/ndt/gfae221)
Supplement: gfae221_Supplemental_File [file gfae221_Supplemental_File.pdf]

Supplementary material

Contents

Table S1. PRISMA checklist

Table S2. Inclusion and exclusion criteria

Table S3: Search strategy for Ovid Medline

Table S4. Criteria for quality assessment for controlled intervention studies

Table S5: Summary of outcomes

Table S6: Clinical outcomes

Table S7: Quality assessment of studies

Figure S2: Forest plots for systolic blood pressure (S2A) and diastolic blood pressure (S2B)

Figure S3: Forest plots for creatinine (S3A) and hemoglobin (S3B)

Table S1. PRISMA 2020 checklist

Section and Topic	Item #	Checklist item	Location where item is reported
**TITLE**			
Title	1	Identify the report as a systematic review.	P1
**ABSTRACT**			
Abstract	2	See the PRISMA 2020 for Abstracts checklist.	P3
**INTRODUCTION**			
Rationale	3	Describe the rationale for the review in the context of existing knowledge.	P6-7
Objectives	4	Provide an explicit statement of the objective(s) or question(s) the review addresses.	P7
**METHODS**			
Eligibility criteria	5	Specify the inclusion and exclusion criteria for the review and how studies were grouped for the syntheses.	P5 – supplementary material
Information sources	6	Specify all databases, registers, websites, organisations, reference lists and other sources searched or consulted to identify studies. Specify the date when each source was last searched or consulted.	P8
Search strategy	7	Present the full search strategies for all databases, registers and websites, including any filters and limits used.	P6-14 supplementary material
Selection process	8	Specify the methods used to decide whether a study met the inclusion criteria of the review, including how many reviewers screened each record and each report retrieved, whether they worked independently, and if applicable, details of automation tools used in the process.	P8
Data collection process	9	Specify the methods used to collect data from reports, including how many reviewers collected data from each report, whether they worked independently, any processes for obtaining or confirming data from study investigators, and if applicable, details of automation tools used in the process.	P9
Data items	10a	List and define all outcomes for which data were sought. Specify whether all results that were compatible with each outcome domain in each study were sought (e.g. for all measures, time points, analyses), and if not, the methods used to decide which results to collect.	P9
	10b	List and define all other variables for which data were sought (e.g. participant and intervention characteristics, funding sources). Describe any assumptions made about any missing or unclear information.	P9
Study risk of bias assessment	11	Specify the methods used to assess risk of bias in the included studies, including details of the tool(s) used, how many reviewers assessed each study and whether they worked independently, and if applicable, details of automation tools used in the process.	P9
Effect measures	12	Specify for each outcome the effect measure(s) (e.g. risk ratio, mean difference) used in the synthesis or presentation of results.	P10
Synthesis methods	13a	Describe the processes used to decide which studies were eligible for each synthesis (e.g. tabulating the study intervention characteristics and comparing against the planned groups for each synthesis (item #5)).	P5 – supplementary

Section and Topic	Item #	Checklist item	Location where item is reported
			material
	13b	Describe any methods required to prepare the data for presentation or synthesis, such as handling of missing summary statistics, or data conversions.	N/A
	13c	Describe any methods used to tabulate or visually display results of individual studies and syntheses.	P10
	13d	Describe any methods used to synthesize results and provide a rationale for the choice(s). If meta-analysis was performed, describe the model(s), method(s) to identify the presence and extent of statistical heterogeneity, and software package(s) used.	P10
	13e	Describe any methods used to explore possible causes of heterogeneity among study results (e.g. subgroup analysis, meta-regression).	P10
	13f	Describe any sensitivity analyses conducted to assess robustness of the synthesized results.	P10
Reporting bias assessment	14	Describe any methods used to assess risk of bias due to missing results in a synthesis (arising from reporting biases).	N/A
Certainty assessment	15	Describe any methods used to assess certainty (or confidence) in the body of evidence for an outcome.	N/A
**RESULTS**			
Study selection	16a	Describe the results of the search and selection process, from the number of records identified in the search to the number of studies included in the review, ideally using a flow diagram.	Page 1 in "Figures" document
	16b	Cite studies that might appear to meet the inclusion criteria, but which were excluded, and explain why they were excluded.	N/A
Study characteristics	17	Cite each included study and present its characteristics.	P35-51
Risk of bias in studies	18	Present assessments of risk of bias for each included study.	Supplementary material – P37
Results of individual studies	19	For all outcomes, present, for each study: (a) summary statistics for each group (where appropriate) and (b) an effect estimate and its precision (e.g. confidence/credible interval), ideally using structured tables or plots.	P13-14, 52-60
Results of syntheses	20a	For each synthesis, briefly summarise the characteristics and risk of bias among contributing studies.	P11-12
	20b	Present results of all statistical syntheses conducted. If meta-analysis was done, present for each the summary estimate and its precision (e.g. confidence/credible interval) and measures of statistical heterogeneity. If comparing groups, describe the direction of the effect.	P13-14
	20c	Present results of all investigations of possible causes of heterogeneity among study results.	P13-14
	20d	Present results of all sensitivity analyses conducted to assess the robustness of the synthesized results.	P13-14
Reporting biases	21	Present assessments of risk of bias due to missing results (arising from reporting biases) for each synthesis assessed.	N/A
Certainty of evidence	22	Present assessments of certainty (or confidence) in the body of evidence for each outcome assessed.	N/A

Section and Topic	Item #	Checklist item	Location where item is reported
**DISCUSSION**			
Discussion	23a	Provide a general interpretation of the results in the context of other evidence.	P16-22
	23b	Discuss any limitations of the evidence included in the review.	P18-19
	23c	Discuss any limitations of the review processes used.	P19-20
	23d	Discuss implications of the results for practice, policy, and future research.	P20-21
**OTHER INFORMATION**			
Registration and protocol	24a	Provide registration information for the review, including register name and registration number, or state that the review was not registered.	P23
	24b	Indicate where the review protocol can be accessed, or state that a protocol was not prepared.	P8
	24c	Describe and explain any amendments to information provided at registration or in the protocol.	N/A
Support	25	Describe sources of financial or non-financial support for the review, and the role of the funders or sponsors in the review.	P23
Competing interests	26	Declare any competing interests of review authors.	P23
Availability of data, code and other materials	27	Report which of the following are publicly available and where they can be found: template data collection forms; data extracted from included studies; data used for all analyses; analytic code; any other materials used in the review.	P23

From: Page MJ, McKenzie JE, Bossuyt PM, Boutron I, Hoffmann TC, Mulrow CD, et al. The PRISMA 2020 statement: an updated guideline for reporting systematic reviews. BMJ 2021;372:n71. doi: 10.1136/bmj.n71

Table S2. Inclusion and exclusion criteria

Inclusion criteria (PICOD)	Exclusion criteria
• Population: Adults that are aged 18 years or over with a diagnosis of CKD, including those with non-dialysis CKD and those receiving (in recipient of) kidney replacement therapy, such as dialysis or transplantation.<br>• Intervention: Any intervention delivered by a pharmacist (e.g., patient counselling)<br>• Comparator: Any appropriate comparator group (e.g., usual care).<br>• Outcomes: Any economic, clinical, or humanistic outcome measures.<br>*Design: Any form of RCTs, including clustered controlled trials and any trial where participants are randomized to receive an intervention(s). This includes wait-list or cross-over study designs.*	• Abstracts/conference proceedings<br>• Protocols<br>• Seminars<br>• Case-control studies<br>• Case reports<br>• Cohort studies<br>• Case series<br>• Cross-sectional studies<br>• Adolescents and children below the age of 18 years and animals<br>• Non-English studies

Abbreviations: CKD, Chronic kidney disease; e.g., Exempli gratia; RCT, Randomized controlled trial

Table S3: Search strategy for Ovid Medline

1. pharmacist*.mp. [mp=title, abstract, original title, name of substance word, subject heading word, floating sub-heading word, keyword heading word, organism supplementary concept word, protocol supplementary concept word, rare disease supplementary concept word, unique identifier, synonyms]
2. intervention*.mp. [mp=title, abstract, original title, name of substance word, subject heading word, floating sub-heading word, keyword heading word, organism supplementary concept word, protocol supplementary concept word, rare disease supplementary concept word, unique identifier, synonyms]
3. Medic* management.mp. [mp=title, abstract, original title, name of substance word, subject heading word, floating sub-heading word, keyword heading word, organism supplementary concept word, protocol supplementary concept word, rare disease supplementary concept word, unique identifier, synonyms]
4. Medic* reconciliation.mp. [mp=title, abstract, original title, name of substance word, subject heading word, floating sub-heading word, keyword heading word, organism supplementary concept word, protocol supplementary concept word, rare disease supplementary concept word, unique identifier, synonyms]
5. Medic* adherence.mp. [mp=title, abstract, original title, name of substance word, subject heading word, floating sub-heading word, keyword heading word, organism supplementary concept word, protocol supplementary concept word, rare disease supplementary concept word, unique identifier, synonyms]
6. Medic*optimi?ation.mp. [mp=title, abstract, original title, name of substance word, subject heading word, floating sub-heading word, keyword heading word, organism supplementary concept word, protocol supplementary concept word, rare disease supplementary concept word, unique identifier, synonyms]
7. Medic* review.mp. [mp=title, abstract, original title, name of substance word, subject heading word, floating sub-heading word, keyword heading word, organism supplementary concept word, protocol supplementary concept word, rare disease supplementary concept word, unique identifier, synonyms]

8. Medic* monitoring.mp. [mp=title, abstract, original title, name of substance word, subject heading word, floating sub-heading word, keyword heading word, organism supplementary concept word, protocol supplementary concept word, rare disease supplementary concept word, unique identifier, synonyms]
9. Lifestyle advice.mp. [mp=title, abstract, original title, name of substance word, subject heading word, floating sub-heading word, keyword heading word, organism supplementary concept word, protocol supplementary concept word, rare disease supplementary concept word, unique identifier, synonyms]
10. Life style advice.mp. [mp=title, abstract, original title, name of substance word, subject heading word, floating sub-heading word, keyword heading word, organism supplementary concept word, protocol supplementary concept word, rare disease supplementary concept word, unique identifier, synonyms]
11. Patient education.mp. [mp=title, abstract, original title, name of substance word, subject heading word, floating sub-heading word, keyword heading word, organism supplementary concept word, protocol supplementary concept word, rare disease supplementary concept word, unique identifier, synonyms]
12. Patient counsel?ing.mp. [mp=title, abstract, original title, name of substance word, subject heading word, floating sub-heading word, keyword heading word, organism supplementary concept word, protocol supplementary concept word, rare disease supplementary concept word, unique identifier, synonyms]
13. Pharmacotherap*.mp. [mp=title, abstract, original title, name of substance word, subject heading word, floating sub-heading word, keyword heading word, organism supplementary concept word, protocol supplementary concept word, rare disease supplementary concept word, unique identifier, synonyms]
14. Drug therap*.mp. [mp=title, abstract, original title, name of substance word, subject heading word, floating sub-heading word, keyword heading word, organism supplementary concept word, protocol supplementary concept word, rare disease supplementary concept word, unique identifier, synonyms]
15. Motivational interview*.mp. [mp=title, abstract, original title, name of substance word, subject heading word, floating sub-heading word, keyword heading word, organism supplementary concept word, protocol supplementary concept word, rare disease supplementary concept word, unique identifier, synonyms]

16. Non-pharmaceutical.mp. [mp=title, abstract, original title, name of substance word, subject heading word, floating sub-heading word, keyword heading word, organism supplementary concept word, protocol supplementary concept word, rare disease supplementary concept word, unique identifier, synonyms]
17. Nonpharmaceutical.mp. [mp=title, abstract, original title, name of substance word, subject heading word, floating sub-heading word, keyword heading word, organism supplementary concept word, protocol supplementary concept word, rare disease supplementary concept word, unique identifier, synonyms]
18. Smoking cessation.mp. [mp=title, abstract, original title, name of substance word, subject heading word, floating sub-heading word, keyword heading word, organism supplementary concept word, protocol supplementary concept word, rare disease supplementary concept word, unique identifier, synonyms]
19. Self-management.mp. [mp=title, abstract, original title, name of substance word, subject heading word, floating sub-heading word, keyword heading word, organism supplementary concept word, protocol supplementary concept word, rare disease supplementary concept word, unique identifier, synonyms]
20. Deprescribing.mp. [mp=title, abstract, original title, name of substance word, subject heading word, floating sub-heading word, keyword heading word, organism supplementary concept word, protocol supplementary concept word, rare disease supplementary concept word, unique identifier, synonyms]
21. Polypharmacy.mp. [mp=title, abstract, original title, name of substance word, subject heading word, floating sub-heading word, keyword heading word, organism supplementary concept word, protocol supplementary concept word, rare disease supplementary concept word, unique identifier, synonyms]
22. Chronic kidney disease.mp. [mp=title, abstract, original title, name of substance word, subject heading word, floating sub-heading word, keyword heading word, organism supplementary concept word, protocol supplementary concept word, rare disease supplementary concept word, unique identifier, synonyms]
23. CKD.mp. [mp=title, abstract, original title, name of substance word, subject heading word, floating sub-heading word, keyword heading word, organism supplementary concept word, protocol supplementary concept word, rare disease supplementary concept word, unique identifier, synonyms]

24. Chronic renal disease.mp. [mp=title, abstract, original title, name of substance word, subject heading word, floating sub-heading word, keyword heading word, organism supplementary concept word, protocol supplementary concept word, rare disease supplementary concept word, unique identifier, synonyms]
25. CRD.mp. [mp=title, abstract, original title, name of substance word, subject heading word, floating sub-heading word, keyword heading word, organism supplementary concept word, protocol supplementary concept word, rare disease supplementary concept word, unique identifier, synonyms]
26. Renal insufficiency.mp. [mp=title, abstract, original title, name of substance word, subject heading word, floating sub-heading word, keyword heading word, organism supplementary concept word, protocol supplementary concept word, rare disease supplementary concept word, unique identifier, synonyms]
27. Kidney insufficiency.mp. [mp=title, abstract, original title, name of substance word, subject heading word, floating sub-heading word, keyword heading word, organism supplementary concept word, protocol supplementary concept word, rare disease supplementary concept word, unique identifier, synonyms]
28. Chronic renal insufficiency.mp. [mp=title, abstract, original title, name of substance word, subject heading word, floating sub-heading word, keyword heading word, organism supplementary concept word, protocol supplementary concept word, rare disease supplementary concept word, unique identifier, synonyms]
29. Chronic kidney insufficiency.mp. [mp=title, abstract, original title, name of substance word, subject heading word, floating sub-heading word, keyword heading word, organism supplementary concept word, protocol supplementary concept word, rare disease supplementary concept word, unique identifier, synonyms]
30. Renal impairment.mp. [mp=title, abstract, original title, name of substance word, subject heading word, floating sub-heading word, keyword heading word, organism supplementary concept word, protocol supplementary concept word, rare disease supplementary concept word, unique identifier, synonyms]
31. Kidney impairment.mp. [mp=title, abstract, original title, name of substance word, subject heading word, floating sub-heading word, keyword heading word, organism supplementary concept word, protocol supplementary concept word, rare disease supplementary concept word, unique identifier, synonyms]

32. Renal replacement therapy.mp. [mp=title, abstract, original title, name of substance word, subject heading word, floating sub-heading word, keyword heading word, organism supplementary concept word, protocol supplementary concept word, rare disease supplementary concept word, unique identifier, synonyms]
33. RRT.mp. [mp=title, abstract, original title, name of substance word, subject heading word, floating sub-heading word, keyword heading word, organism supplementary concept word, protocol supplementary concept word, rare disease supplementary concept word, unique identifier, synonyms]
34. Kidney replacement therapy.mp. [mp=title, abstract, original title, name of substance word, subject heading word, floating sub-heading word, keyword heading word, organism supplementary concept word, protocol supplementary concept word, rare disease supplementary concept word, unique identifier, synonyms]
35. KRT.mp. [mp=title, abstract, original title, name of substance word, subject heading word, floating sub-heading word, keyword heading word, organism supplementary concept word, protocol supplementary concept word, rare disease supplementary concept word, unique identifier, synonyms]
36. H?emodialysis.mp. [mp=title, abstract, original title, name of substance word, subject heading word, floating sub-heading word, keyword heading word, organism supplementary concept word, protocol supplementary concept word, rare disease supplementary concept word, unique identifier, synonyms]
37. HD.mp. [mp=title, abstract, original title, name of substance word, subject heading word, floating sub-heading word, keyword heading word, organism supplementary concept word, protocol supplementary concept word, rare disease supplementary concept word, unique identifier, synonyms]
38. Peritoneal dialysis.mp. [mp=title, abstract, original title, name of substance word, subject heading word, floating sub-heading word, keyword heading word, organism supplementary concept word, protocol supplementary concept word, rare disease supplementary concept word, unique identifier, synonyms]
39. PD.mp. [mp=title, abstract, original title, name of substance word, subject heading word, floating sub-heading word, keyword heading word, organism supplementary concept word, protocol supplementary concept word, rare disease supplementary concept word, unique identifier, synonyms]

40. Kidney dialysis.mp. [mp=title, abstract, original title, name of substance word, subject heading word, floating sub-heading word, keyword heading word, organism supplementary concept word, protocol supplementary concept word, rare disease supplementary concept word, unique identifier, synonyms]
41. Renal dialysis.mp. [mp=title, abstract, original title, name of substance word, subject heading word, floating sub-heading word, keyword heading word, organism supplementary concept word, protocol supplementary concept word, rare disease supplementary concept word, unique identifier, synonyms]
42. Kidney failure.mp. [mp=title, abstract, original title, name of substance word, subject heading word, floating sub-heading word, keyword heading word, organism supplementary concept word, protocol supplementary concept word, rare disease supplementary concept word, unique identifier, synonyms]
43. Renal failure.mp. [mp=title, abstract, original title, name of substance word, subject heading word, floating sub-heading word, keyword heading word, organism supplementary concept word, protocol supplementary concept word, rare disease supplementary concept word, unique identifier, synonyms]
44. Kidney transplant*.mp. [mp=title, abstract, original title, name of substance word, subject heading word, floating sub-heading word, keyword heading word, organism supplementary concept word, protocol supplementary concept word, rare disease supplementary concept word, unique identifier, synonyms]
45. Renal transplant*.mp. [mp=title, abstract, original title, name of substance word, subject heading word, floating sub-heading word, keyword heading word, organism supplementary concept word, protocol supplementary concept word, rare disease supplementary concept word, unique identifier, synonyms]
46. End-stage kidney disease.mp. [mp=title, abstract, original title, name of substance word, subject heading word, floating sub-heading word, keyword heading word, organism supplementary concept word, protocol supplementary concept word, rare disease supplementary concept word, unique identifier, synonyms]
47. ESKD.mp. [mp=title, abstract, original title, name of substance word, subject heading word, floating sub-heading word, keyword heading word, organism supplementary concept word, protocol supplementary concept word, rare disease supplementary concept word, unique identifier, synonyms]

48.	End-stage renal disease.mp. [mp=title, abstract, original title, name of substance word, subject heading word, floating sub-heading word, keyword heading word, organism supplementary concept word, protocol supplementary concept word, rare disease supplementary concept word, unique identifier, synonyms]
49.	ESRD.mp. [mp=title, abstract, original title, name of substance word, subject heading word, floating sub-heading word, keyword heading word, organism supplementary concept word, protocol supplementary concept word, rare disease supplementary concept word, unique identifier, synonyms]
50.	exp Early Intervention, Educational/ or exp Early Medical Intervention/
51.	exp Medication Therapy Management/
52.	exp Medication Reconciliation/
53.	exp Medication Adherence/
54.	exp Kidney Failure, Chronic/ or exp Renal Dialysis/ or exp Renal Insufficiency, Chronic/ or exp Kidney Transplantation/
55.	exp Patient Education as Topic/
56.	exp Counseling/
57.	exp Drug Therapy/
58.	exp Motivational Interviewing/
59.	exp Smoking Cessation/
60.	exp Self Care/ or exp Self-Management/
61.	exp Deprescriptions/
62.	exp Polypharmacy/ or exp Drug Therapy, Combination/

63.	exp Renal Insufficiency/
64.	exp Renal Replacement Therapy/
65.	exp Peritoneal Dialysis/
66.	exp Pharmacy Service, Hospital/ or exp Pharmacists/ or exp Pharmacies/ or exp Community Pharmacy Services/
67.	exp Kidney Diseases/
68.	exp Life Style/
69.	dos* adjust*.mp. [mp=title, abstract, original title, name of substance word, subject heading word, floating sub-heading word, keyword heading word, organism supplementary concept word, protocol supplementary concept word, rare disease supplementary concept word, unique identifier, synonyms]
70.	exp Intersectoral Collaboration/ or exp Interprofessional Relations/ or exp Interdisciplinary Communication/
71.	collaborati*.mp. [mp=title, abstract, original title, name of substance word, subject heading word, floating sub-heading word, keyword heading word, organism supplementary concept word, protocol supplementary concept word, rare disease supplementary concept word, unique identifier, synonyms]
72.	pharmaceutical care.mp. [mp=title, abstract, original title, name of substance word, subject heading word, floating sub-heading word, keyword heading word, organism supplementary concept word, protocol supplementary concept word, rare disease supplementary concept word, unique identifier, synonyms]
73.	exp Pharmaceutical Services/
74.	exp Drug Monitoring/
75.	drug monitor*.mp. [mp=title, abstract, original title, name of substance word, subject heading word, floating sub-heading word, keyword heading word, organism supplementary concept word, protocol supplementary concept word, rare disease supplementary concept word, unique identifier, synonyms]

76.	multi-disciplinary.mp. [mp=title, abstract, original title, name of substance word, subject heading word, floating sub-heading word, keyword heading word, organism supplementary concept word, protocol supplementary concept word, rare disease supplementary concept word, unique identifier, synonyms]
77.	multidisciplinary.mp. [mp=title, abstract, original title, name of substance word, subject heading word, floating sub-heading word, keyword heading word, organism supplementary concept word, protocol supplementary concept word, rare disease supplementary concept word, unique identifier, synonyms]
78.	exp Patient Care Team/
79.	exp Pharmacy/
80.	pharmacy.mp. [mp=title, abstract, original title, name of substance word, subject heading word, floating sub-heading word, keyword heading word, organism supplementary concept word, protocol supplementary concept word, rare disease supplementary concept word, unique identifier, synonyms]
81.	pharmacies.mp. [mp=title, abstract, original title, name of substance word, subject heading word, floating sub-heading word, keyword heading word, organism supplementary concept word, protocol supplementary concept word, rare disease supplementary concept word, unique identifier, synonyms]
82.	exp Pharmacies/
83.	1 or 66 or 79 or 80 or 81 or 82
84.	2 or 3 or 4 or 5 or 6 or 7 or 8 or 9 or 10 or 11 or 12 or 13 or 14 or 15 or 16 or 17 or 18 or 19 or 20 or 21 or 50 or 51 or 52 or 53 or 55 or 56 or 57 or 58 or 59 or 60 or 61 or 62 or 68 or 69 or 70 or 71 or 72 or 73 or 74 or 75 or 76 or 77 or 78
85.	22 or 23 or 24 or 25 or 26 or 27 or 28 or 29 or 30 or 31 or 32 or 33 or 34 or 35 or 36 or 37 or 38 or 39 or 40 or 41 or 42 or 43 or 44 or 45 or 46 or 47 or 48 or 49 or 54 or 63 or 64 or 65 or 67
86.	83 and 84 and 85

Table S4. Criteria for quality assessment for controlled intervention studies

Criteria
1. Was the study described as randomized, a randomized trial, a randomized clinical trial, or an RCT?
2. Was the method of randomization adequate (i.e., use of randomly generated assignment)?
3. Was the treatment allocation concealed (so that assignments could not be predicted)?
4. Were study participants and providers blinded to treatment group assignment?
5. Were the people assessing the outcomes blinded to the participants' group assignments?
6. Were the groups similar at baseline on important characteristics that could affect outcomes (e.g., demographics, risk factors, co-morbid conditions)?
7. Was the overall drop-out rate from the study at endpoint 20% or lower of the number allocated to treatment?
8. Was the differential drop-out rate (between treatment groups) at endpoint 15 percentage points or lower?
9. Was there high adherence to the intervention protocols for each treatment group?
10. Were other interventions avoided or similar in the groups (e.g., similar background treatments)?

11. Were outcomes assessed using valid and reliable measures, implemented consistently across all study participants?

12. Did the authors report that the sample size was sufficiently large to be able to detect a difference in the main outcome between groups with at least 80% power?

13. Were outcomes reported or subgroups analysed prespecified (i.e., identified before analyses were conducted)?

14. Were all randomized participants analysed in the group to which they were originally assigned, i.e., did they use an intention-to-treat analysis?

Abbreviations: e.g., Exempli gratia; i.e., Id est; RCT: Randomized controlled trial

Table S5: Summary of outcomes

Author (year), country	Summary of outcomes
Al Hamarneh (2018), Canada	**Economic:** N/R
	**Clinical:** Estimated CV risk, LDL, SBP, DBP, HbA1C, smoking cessation, 5-year predicted risk for developing ESRD, initiation of ACEI, statins, and ARBs, dose changes and medication changes for diabetes, dyslipidemia, hypertension, and impact of rural vs. urban residence on the difference in change in estimated CV risk
	**Humanistic:** N/R
Alshogran (2022), Jordan	**Economic:** N/R
	**Clinical:** Patients' total adherence to recommendations, HD attendance adherence, diet restriction adherence, duration of shortening HD, episodes of shortening HD, fluid restriction adherence, medication adherence, hospital and emergency room admissions after follow-up, and levels of sodium, potassium, urea, creatinine, albumin, calcium, corrected calcium, phosphorus, calcium*phosphorus product, hemoglobin, and PTH
	**Humanistic:** Patients' perception of adherence behaviors, disease awareness, and HRQoL
Armstrong (2000), United States	**Economic:** N/R
	**Clinical:** Serum ferritin and/or transferrin saturation, hematocrit measurements, adverse events, effective treatment of anemia
	**Humanistic:** N/R
Bessa (2016), Brazil	**Economic**: N/R
	**Clinical:** %CV, mean %CV, % of patients who achieved TAC target concentrations in each study visit, comparison of mean dose-corrected whole blood TAC trough concentrations from day 7 to day 90, incidence of infections, acute rejection, eGFR, death, graft loss, discontinuation of immunosuppressive treatment, hospital readmissions, medication adherence
	**Humanistic:** N/R
Bhardwaja (2011), United States	**Economic**: N/R
	**Clinical**: Proportion of medication errors
	**Humanistic**: N/R
Chang (2016), United States	**Economic**: N/R
	**Clinical**: Proteinuria screening and lipid screening within 1 year of the enrolment date, achieved BP goal (<140/90 mmHg for non-proteinuric CKD and <130/80 mmHg for proteinuric CKD), proportion of proteinuric CKD patients taking an ACEI or ARB, proportion of patients on statins
	**Humanistic**: Patient survey on acceptability of pharmacist MTM
Chisholm-Burns (2013), United States	**Economic**: Cost-saving
	**Clinical**: Immunosuppressant adherence, days in hospital, emergency department visits, outpatient visits and homecare visits
	**Humanistic**: N/R
Chisholm (2001), United States	**Economic**: N/R
	**Clinical**: Immunosuppressant compliance rates, patterns of compliance, and serum immunosuppressant concentrations
	**Humanistic**: N/R
Chisholm (2002), United States	**Economic**: N/R
	**Clinical**: SBP and DBP
	**Humanistic**: N/R
Cohen (2020), United States	**Economic**: N/R
	**Clinical**: % of patients with inadequate MR determined by any one error in MR, the number of medication errors, of all medications and high-risk medications, identified per patient sample
	**Humanistic**: N/R
Cooney (2015), United States	**Economic**: N/R
	**Clinical**: SBP among participants with baseline BP >130/80mmHg, PTH measured during study period, BP < 130/80 mmHg, incidence of ESRD, death, measurement of phosphorus and UACR; the number of antihypertensive medications prescribed to those with poorly controlled hypertension; appropriate treatment with ACEI/ARB, phosphorus binders, Vitamin D, and sodium bicarbonate; medication adherence; and the percent of subjects seen by nephrology
	**Humanistic**: HRQoL and patient satisfaction

Cypes (2021), United States	**Economic**: N/R
	**Clinical**: Number of medications requiring pharmacist intervention and incorrect CKD staging
	**Humanistic**: N/R
Dashti-Khavidaki (2013), Iran	**Economic**: N/R
	**Clinical**: N/R
	**Humanistic:** HRQoL
Fleming (2021), United States	**Economic**: N/R
	**Clinical**: Mean TAC intrapatient variability from baseline to 12 months post randomization and proportion of patients achieving TAC intrapatient variability of <30% and <40% at end of study
	**Humanistic**: Patient feedback on app
Gonzales (2021), United States	**Economic**: N/R
	**Clinical**: Incidence and severity of medication errors, incidence and severity of adverse events, adverse event rate, hospitalization rate, infection rate and opportunistic infection rate
	**Humanistic**: N/R
Ishani (2016), United States	**Economic**: N/R
	**Clinical**: A composite of death, hospitalization, emergency department visits, and admission to a skilled nursing facility, each component of the composite, and incidence of ESRD
	**Humanistic**: N/R
Lalonde (2017), Canada	**Economic**: N/R
	**Clinical**: Mean number of DRPs, eGFR, SBP, DBP, HbA1C, LDL cholesterol
	**Humanistic**: N/R
Marouf (2020, Iraq	**Economic**: N/R
	**Clinical**: Hemoglobin, TSAT, serum ferritin, serum vitamin B12, serum folate and clinical grading of pallor
	**Humanistic**: N/R
Mateti (2018), India	**Economic**: Utility values and QALY, Cost data, Cost-effectiveness Grid and Plane and ICER
	**Clinical**: SBP, DBP, IDW, hemoglobin levels, survival time and medication adherence rate
	**Humanistic**: HRQoL
Okoro (2022), Nigeria	**Economic:** N/R
	**Clinical:** Changes in BP from baseline to 6 months and 12 months, from 6 to 12 months, proportion of participants with controlled BP to less than 130/80 mmHg at 6 and 12 months, respectively; changes in antihypertensive medication adherence and serum creatinine levels from baseline to 6 and 12 months, and 6 to 12 months, respectively
	**Humanistic:** Patients' satisfaction with care received
Pai (2009), United States	**Economic**: N/R
	**Clinical**: N/R
	**Humanistic**: HRQoL
Pai (2009), United States	**Economic**: Cost of all concomitant drugs
	**Clinical**: Number of medications used, rate of hospitalization, length of hospitalization
	**Humanistic**: N/R
Peralta (2020), United States	**Economic**: N/R
	**Clinical**: SBP, DBP, controlled BP (<140/90 mm Hg), ACEi/ARB use, ACEi/ARB initiation, statin therapy use, statin therapy initiation, diuretic use, and diuretic initiation
	**Humanistic**: N/R
Qudah (2016), Jordan	**Economic**: N/R
	**Clinical**: Patients who reached weekly average home BP target of SBP ≤135 mmHg and DBP≤85 mmHg, absolute changes in average weekly home SBP and DBP measurements at the end of the study, absolute changes in pre-, post-, and intradialysis BP, absolute changes in IDWG, and adherence to antihypertensive therapy
	**Humanistic**: N/R
Quintana-Barcena (2018), Canada	**Economic**: N/R
	**Clinical**: The number and severity of DRPs
	**Humanistic**: N/R
	**Economic**: N/R

Rifkin (2013), United States	**Clinical**: SBP, DBP, mean arterial pressure, serum creatinine levels, eGFR, total number of medications, number of blood pressure medications, data exchange and medication adherence
	**Humanistic**: Participant feedback on device acceptability
Santschi (2011), Canada	**Economic**: N/R
	**Clinical**: SBP, DBP and BP control (<130/80 mmHg), number of antihypertensive drugs and class of antihypertensive medications used
	**Humanistic**: N/R
Sathvik, (2007), India	**Economic**: N/R
	**Clinical**: N/R
	**Humanistic**: Patients' medication knowledge for name of medications, indication, strength, and number of doses to be taken
Skoutakis (1978), United States	**Economic**: N/R
	**Clinical**: Drug compliance and patients' biochemical and therapeutic responses
	**Humanistic**: Patients' knowledge and understanding of renal disease, dialysis procedures, and drug and dietary management
Song (2021), South Korea	**Economic**: N/R
	**Clinical**: Number of DRPs per patient at discharge, DRP classification, medication adherence for discharge drugs, a composite of acute care utilization (unexpected hospitalization or emergency center visit) within 3 months of discharge, and change in the number of unintentional medication discrepancies at discharge compared with that at the time of admission
	**Humanistic**: N/R
Taber (2021), United States	**Economic**: Charges to payer, Multivariable modelling for payer charges and ROI
	**Clinical**: Acute rejections, graft losses, LOS hospitalizations
	**Humanistic: N/R**
Tamilselvan (2021), India	**Economic**: N/R
	**Clinical**: N/R
	**Humanistic**: HRQOL
Theeranut (2021), Thailand	**Economic**: N/R
	**Clinical**: Mean difference of eGFR from baseline, proportion of patients with eGFR decline greater than 4 mL/min/1.73 m2, and difference in CKD stage from baseline
	**Humanistic**: N/R
Tuttle (2018), United States	**Economic**: N/R
	**Clinical**: A composite of acute care utilization events (hospitalization or emergency department and urgent care center visits) for 90 days after hospital discharge, individual events comprising the composite, eGFR, serum creatinine levels, UACR, use rates of ACE inhibitors or ARBs and goals for BP, SBP, DBP, HbA1c (diabetic participants), hemoglobin, phosphorus, and PTH
	**Humanistic**: N/R
Van den Oever (2020), Netherlands	**Economic**: N/R
	**Clinical**: Median percentage of monthly hemoglobin values in the follow-up period that were in PTR, PSTR, the PTR for iron, PBTR, weekly DA dose, patients with mean dose of ≥90 mcg DA per week, iron sucrose dose, all-cause mortality, and number of patients with at least one transfusion during follow-up
	**Humanistic**: N/R
Yokum (2008), United Kingdom	**Economic**: N/R
	**Clinical**: Serum levels of phosphate, calcium-phosphate product, corrected calcium, intact PTH; medication adjustments
	**Humanistic**: N/R

Table S6 - Clinical outcomes

Study	Umbrella outcome domain	Name	Direction of effect	Units/measured by
Gonzales (2021)	Adverse events	adverse event rate	↔	No units. Total(N), per-patient year; adjusted IRR (95%CI). After 12 months.
Armstrong (2000)	Adverse events	Adverse events	↔	No units/%. Measured at 6 and 12 months.
Gonzales (2021)	Adverse events	incidence of adverse events	↔	No units. Total(N) After 12 months.
Gonzales (2021)	Adverse events	severity of adverse events	↔	No units. Total(N) After 12 months.
Cooney (2015)	Appropriate medical treatment	Appropriate treatment with ACEI/ARB	↔	No units. N(%); Measured after one year.
Cooney (2015)	Appropriate medical treatment	Appropriate treatment with phosphorus binders	↔	No units. N(%); Measured after one year.
Cooney (2015)	Appropriate medical treatment	Appropriate treatment with sodium bicarbonate	↑	No units. N(%); Measured after one year.
Cooney (2015)	Appropriate medical treatment	Appropriate treatment with Vitamin D	↑	No units. N(%); Measured after one year.
Chang (2016)	BP	Achieved BP goal (<140/90 for non-proteinuric CKD and <130/80 for proteinuric CKD)	↔	No units/n(%, OR, 95% CI). Measured at end of trial (end of year).

Cooney (2015)	BP	BP < 130/80 mmHg	↔	No units. N(%); Measured after one year.
Santschi (2011)	BP	BP control (<130/80mmHg)	↔	n(%) measured at 6th month. Unadjusted and adjusted relative risks (95% CI).
Peralta (2020)	BP	Controlled BP (<140/90 mm Hg)	↔	For as-treated and intention to treat analyses: No units/n(%). After 12 months.
Al Hamarneh (2018)	BP	DBP	↑	mmHg; mean±SD, difference in change (95% CI). Measured after three months.
Chisholm (2002)	BP	DBP	**↑ for 2nd and 4th quarter; ↔ for 3rd quarter**	mmHg/mean±SD. Measured at 1st, 2nd, 3rd and 4th quarters.
Lalonde (2017)	BP	DBP	↔	mmHg/mean±SD; unadjusted and adjusted incremental mean change (95% confidence interval). Measured after 12 months.
Mateti (2018)	BP	DBP	**↑ for all hospitals**	mmHg/mean±SD; Greenhouse-Geisser F. Measured at 6th and 12 month.
Okoro (2022)	BP	DBP at 6 months	↔	mmHg. Mean(SD); Measured after 6 months.
Okoro (2022)	BP	DBP at 12 months	↔	mmHg. Mean(SD); Measured after 12 months.

Peralta (2020)	BP	DBP	↔	For as-treated and intention to treat analyses: mmHg/mean±SD. After 12 months.
Qudah (2016)	BP	Average weekly home DBP	↔	mmHg/mean±SD. Measured after three months.
Qudah (2016)	BP	absolute changes in pre-dialysis DBP -	↔	mmHg/mean±SD. Measured after three months.
Qudah (2016)	BP	absolute changes in post-dialysis DBP	↔	mmHg/mean±SD. Measured after three months.
Qudah (2016)	BP	absolute changes in intradialysis DBP	↔	mmHg/mean±SD. Measured after three months.
Rifkin (2013)	BP	DBP	↔	mmHg/mean±SD; change - median and IQR. After 6 months.
Santschi (2011)	BP	DBP	↔	mmHg (mean±SD). unadjusted and adjusted changes; no units (mean, 95% CI). Both measured at 6th month.
Tuttle (2018)	BP	DBP	↔	mmHg/mean±SD. Measured at 30 and 90 days.
Tuttle (2018)	BP	goals for BP (either 140/90 or 130/80 mm Hg)	↔	mmHg/n(%); mean±SD. Measured at 30 and 90 days.
Rifkin (2013)	BP	Mean arterial pressure	↔	mmHg/mean±SD; change - median and IQR. After 6 months.
Qudah (2016)	BP	Patients who reached weekly average home BP target of SBP ≤135 mmHg and DBP≤85 mmHg,	↑	No units/n(%); (OR, 95% CI). Measured after three months.
Okoro (2022)	BP	proportion of participants with controlled BP to less than 130/80 mmHg at 12 months	↔	No units. N(%). Measured after 12 months.
Okoro (2022)	BP	Proportion of participants with controlled BP to less than 130/80 mmHg at 6 months	↔	No units. N(%). Measured after 6 months.
Al Hamarneh (2018)	BP	SBP	↑	mmHg; mean±SD, difference in change (95%

				CI). Measured after three months.
Chisholm (2002)	BP	SBP	↑	mmHg/mean±SD. Measured at 1st, 2nd, 3rd and 4th quarters.
Lalonde (2017)	BP	SBP	↔	mmHg/mean±SD; unadjusted and adjusted incremental mean change (95% confidence interval). Measured after 12 months.
Mateti (2018)	BP	SBP	**↑ for all hospitals**	mmHg/mean±SD; Greenhouse-Geisser F. Measured at 6th and 12 month.
Okoro (2022)	BP	SBP at 6 months	↑	mmHg. Mean(SD); Measured after 6 months.
Okoro (2022)	BP	SBP at 12 months	↔	mmHg. Mean(SD); Measured after 12 months.
Peralta (2020)	BP	SBP	↔	For as-treated and intention to treat analyses: mmHg/mean±SD. After 12 months.
Qudah (2016)	BP	Average weekly home SBP	↑	mmHg/mean±SD. Measured after three months.
Qudah (2016)	BP	absolute changes in pre-dialysis SBP	↔	mmHg/mean±SD. Measured after three months.
Qudah (2016)	BP	absolute changes in post-dialysis SBP	↔	mmHg/mean±SD. Measured after three months.
Qudah (2016)	BP	absolute changes in intradialysis SBP	↔	mmHg/mean±SD. Measured after three months.
Rifkin (2013)	BP	SBP	↔	mmHg/mean±SD; change - median and IQR. After 6 months.
Santschi (2011)	BP	SBP	↑	mmHg (mean±SD). unadjusted and adjusted changes; no units (mean,

				95% CI). Both measured at 6th month.
Tuttle (2018)	BP	SBP	↔	mmHg/mean±SD. Measured at 30 and 90 days.
Cooney (2015)	BP	SBP among participants with baseline BP >130/80mmHg	↔	mmHg. Mean(SD); Measured after one year.
Alshogran (2022)	Clinical blood marker	Albumin level	↔	g/dL; mean±SD. Measured three months later.
Alshogran (2022)	Clinical blood marker	Calcium level	↔	mg/dL; mean±SD. Measured three months later
Alshogran (2022)	Clinical blood marker	Calcium*phosphorus product level	↔	mg2/dL2; mean±SD. Measured three months later.
Yokum (2008)	Clinical blood marker	calcium-phosphate product levels	↑	mmol2/l2 (mean ± SD; n(%)). After four months.
Alshogran (2022)	Clinical blood marker	Corrected calcium level	↔	mg/dL; mean±SD. Measured three months later
Yokum (2008)	Clinical blood marker	corrected calcium levels	↔	mmol/l (mean ± SD; n(%)). After four months.
Tuttle (2018)	Clinical blood marker	goals for hemoglobin	↔	No units/n(%); mean±SD. Measured at 30 and 90 days.
Tuttle (2018)	Clinical blood marker	goals for HbA1C (diabetic participants)	↔	%/n(%); mean±SD. Measured at 30 and 90 days.
Tuttle (2018)	Clinical blood marker	goals for phosphorous	↔	mg/dl/n(%); mean±SD. Measured at 30 and 90 days.
Tuttle (2018)	Clinical blood marker	goals for PTH	↔	pg/ml/ median (IQR); n(%). Measured at 30 and 90 days.
Armstrong (2000)	Clinical blood marker	Hematocrit measurements	↔	No units/%. Measured at 6 and 12 months.
Marouf (2020)	Clinical blood marker	Hemoglobin	↑	g/dl/mean±SD. Measured at 1st month, 2nd and 4th month.
Alshogran (2022)	Clinical blood marker	Hemoglobin level	↑	g/dL; mean±SD. Measured three months later.

Mateti (2018)	Clinical blood marker	Hemoglobin levels	**↑ for academic and government hospitals; ↔ for corporate hospital**	g/dL/mean±SD; Greenhouse-Geisser F. Measured at 6th and 12 month.
Al Hamarneh (2018)	Clinical blood marker	HbA1C	↑	%; mean±SD, difference in change (95% CI). Measured after three months.
Lalonde (2017)	Clinical blood marker	HbA1C	↔	%/mean±SD; unadjusted and adjusted incremental mean change (95% confidence interval). Measured after 12 months.
Yokum (2008)	Clinical blood marker	intact PTH levels	↔	pmol/l (median (range); n(%)). After four months.
Al Hamarneh (2018)	Clinical blood marker	LDL	↑	mmol/L; mean±SD, difference in change (95% CI). Measured after three months.
Lalonde (2017)	Clinical blood marker	LDL cholesterol	↔	mmol/L/mean±SD; unadjusted and adjusted incremental mean change (95% confidence interval). Measured after 12 months.
Cooney (2015)	Clinical blood marker	Measurement of phosphorus	↑	No units. N(%); Measured after one year.
Van den Oever (2020)	Clinical blood marker	PBTR for hemoglobin	↔	%/median (IQR). End of three monrhs.
Alshogran (2022)	Clinical blood marker	Phosphorus level	↑	mg/dL; mean±SD. Measured three months later
Alshogran (2022)	Clinical blood marker	Potassium level	↔	mmol/L; mean±SD. Measured three months later.
Van den Oever (2020)	Clinical blood marker	PSTR for hemoglobin	↑	%/median (IQR). End of three monrhs.
Alshogran (2022)	Clinical blood marker	PTH level	↔	pg/mL; mean±SD. Measured three months later.

Cooney (2015)	Clinical blood marker	PTH measured during study period	↑	No units. N(%); Measured after one year.
Van den Oever (2020)	Clinical blood marker	PTR for hemoglobin	↑	%/median (IQR). End of three monrhs.
Van den Oever (2020)	Clinical blood marker	PTR for iron	↑	%/median (IQR). End of three monrhs.
Armstrong (2000)	Clinical blood marker	Serum ferritin	↔	No units/%. Measured at 6 and 12 months.
Marouf (2020)	Clinical blood marker	Serum ferritin	↔	ng/ml/mean±SD. Measured at 1st month, 2nd and 4th month.
Marouf (2020)	Clinical blood marker	serum folate	↔	ng/ml/mean±SD. Measured at 1st month, 2nd and 4th month.
Yokum (2008)	Clinical blood marker	Serum phosphate levels	↑	mmol/l (mean ± SD; n(%)). After four months.
Armstrong (2000)	Clinical blood marker	Serum transferrin saturation (TSAT)	↔	No units/%. Measured at 6 and 12 months.
Marouf (2020)	Clinical blood marker	serum vitamin B12	↔	ng/ml/mean±SD. Measured at 1st month, 2nd and 4th month.
Alshogran (2022)	Clinical blood marker	Sodium level	↔	mmol/L; mean±SD. Measured three months later.
Marouf (2020)	Clinical blood marker	TSAT	↑	%/mean±SD. Measured at 1st month, 2nd and 4th month.
Alshogran (2022)	Clinical blood marker	Urea level	↑	mg/dL; mean±SD. Measured three months later
Marouf (2020)	Clinical grading of pallor	Clinical grading of pallor	**↑ (no p-value)**	Mild, moderate, or severe/n(%). Measured at 2nd, 3rd, and 4th month.
Armstrong (2000)	Comorbidity management	Effective treatment of anemia	↔	No units/%. Measured at 6 and 12 months.
Chisholm (2001)	Compliance pattern	Patterns of compliance	↑	%/mean (measured at 12 months post-transplant).

Song (2021)	Composite of adverse events	A composite of acute care utilization (unexpected hospitalization or emergency centre visit)	↔	No units/n(%); (OR, 95% CI); within 3 months of discharge.
Tuttle (2018)	Composite of adverse events	A composite of acute care utilization events (hospitalization and emergency department and urgent care centre visits)	↔	No units/n(%). Within 90 days.
Ishani (2016)	Composite of adverse events	A composite of death, hospitalization, emergency department visits, and admission to a skilled nursing facility,	↔	No units/ n(%) (HR(95% CI)). After one year.
Al Hamarneh (2018)	CV risk	Estimated CV risk	↑	%; mean±SD, relative reduction and absolute difference (95%CI). Measured after three months.
Al Hamarneh (2018)	CV risk	Impact of rural vs. urban residence on the difference in change in estimated CV risk	↔	No units; absoltue difference (95% CI). Measured after three months.
Rifkin (2013)	Data exchange	Data exchange	↑ **(no p-value)**	Number of readings per month (average over six months); median (IQR) per month.
Alshogran (2022)	Dialysis adherence	Diet restriction adherence	↑	No units; mean±SD. Measured three months later.
Alshogran (2022)	Dialysis adherence	Duration of shortening HD	↑	No units; mean±SD. Measured three months later.
Alshogran (2022)	Dialysis adherence	Episodes of shortening HD	↑	No units; mean±SD. Measured three months later.
Alshogran (2022)	Dialysis adherence	Fluid restriction adherence	↑	No units; mean±SD. Measured three months later.

Alshogran (2022)	Dialysis adherence	HD attendance adherence	↔	No units; mean±SD. Measured three months later.
Alshogran (2022)	Dialysis adherence	Patients' total adherence to recommendations	↑	No units; mean±SD. Measured three months later.
Al Hamarneh (2018)	Dose adjustment	Dose changes/no. of patients for diabetes	↔	No units; n(%). Measured after three months.
Al Hamarneh (2018)	Dose adjustment	Dose changes/no. of patients for dyslipidemia	↔	No units; n(%). Measured after three months.
Al Hamarneh (2018)	Dose adjustment	Dose changes/no. of patients for hypertension	↑	No units; n(%). Measured after three months.
Song (2021)	DRPs	DRP classification	↔	No units/n(%); time point not stated.
Lalonde (2017)	DRPs	Mean number of DRPs	↑	No units/mean±SD; unadjusted and adjusted incremental mean change (95% confidence interval). Measured after 12 months.
Quintana-Barcena (2018)	DRPs	Number and severity of DRPs	↔	No units. n/DRPs per patient (mean (SD); incremental change - adjusted and unadjusted changed (mean 95% CI). Measured after 12 months.
Song (2021)	DRPs	Number of DRPs per patient at discharge	↑	No units/mean±SD; time point not stated.
Bessa (2016)	Graft function	Acute rejection	↔	No units. n(%). Measured after 90 days.
Taber (2021)	Graft function	Acute rejections	**↑ (no p-value)**	No units/n(%) (during 12 month study)
Bessa (2016)	Graft function	Graft loss	↔	No units. n(%). Measured after 90 days.
Taber (2021)	Graft function	Graft losses	**↑ (no p-value)**	No units/n(%) (during 12 month study)
Ishani (2016)	Healthcare utilization	admission to a skilled nursing facility	↔	No units/ n(%) (HR(95% CI)). After one year.

Tuttle (2018)	Healthcare utilization	Emergency department and urgent care center visits) for 90 days after hospital discharge,	↔	No units/n(%). Within 90 days.
Chisholm-Burns (2013)	Healthcare utilization	Emergency department visits	↔	No units, mean±SD. Monthly average during one year study period.
Ishani (2016)	Healthcare utilization	emergency department visits	↔	No units/ n(%) (HR(95% CI)). After one year.
Chisholm-Burns (2013)	Healthcare utilization	Homecare visits	↔	No units, mean±SD. Monthly average during one year study period.
Chisholm-Burns (2013)	Healthcare utilization	Outpatient visits	↔	No units, mean±SD. Monthly average during one year study period.
Cooney (2015)	Healthcare utilization	Seen by nephrology	↔	No units. N(%); Measured after one year.
Chisholm-Burns (2013)	Hospitalization	Days in hospital	↑	Days, mean±SD (Monthly average during one year study period); % (at least 1 day in hospital during study period); not hospitalized during study period (RR, 95% CI).
Alshogran (2022)	Hospitalization	hospital and emergency room admissions after follow-up	↑	No units; mean±SD and total number (n). Measured three months later.
Bessa (2016)	Hospitalization	Hospital readmissions	↔	No units. n(%). Measured after 90 days.
Ishani (2016)	Hospitalization	hospitalization	↔	No units/ n(%) (HR(95% CI)). After one year.
Tuttle (2018)	Hospitalization	Hospitalization	↔	No units/n(%). Within 90 days.
Gonzales (2021)	Hospitalization	hospitalization rate	↑	No units. Per-patient year (95% CI) After 12 months.
Pai (2009)	Hospitalization	length of hospitalization	↔	Days/mean±SD (measured during study)

Taber (2021)	Hospitalization	LOS hospitalizations	↔	Days/mean±SD; (RR,95%CI). During 12 month study.
Pai (2009)	Hospitalization	rate of hospitalization	↑	Number of hospitalizations/ mean±SD (measured during study)
Qudah (2016)	IDW	absolute changes in IDWG	↔	%/mean±SD. Measured after three months.
Mateti (2018)	IDW	IDW	**↑ for all hospitals**	L/mean±SD; Greenhouse-Geisser F. Measured at 6th and 12 month.
Bessa (2016)	Immunosuppressant concentration	% of patients who achieved Tacrolimus (TAC) target concentrations in each study visit	↔	No units, n(%). Measured at days 7, 10, 14, 21, 28, 60, and 90
Bessa (2016)	Immunosuppressant concentration	Comparison of mean dose-corrected whole blood TAC trough concentrations from day 7 to day 90	↔	ng/mL. mean±SD. Measured at days 7, 10, 14, 21, 28, 60, and 90
Chisholm (2001)	Immunosuppressant concentration	Serum immunosuppressant concentrations	↑	ng/ml/(%) (not reported when outcome was measured).
Cypes (2021)	Incorrect CKD staging	incorrect CKD staging	↔	No units/n(%), over a three month period.
Bessa (2016)	Infection	Incidence of infections	↔	No units. n(%). Measured after 90 days.
Gonzales (2021)	Infection	infection rate	↔	No units. Per-patient year (95% CI)After 12 months.
Gonzales (2021)	Infection	Opportunistic infection rate	↔	No units. Per-patient year (95% CI)After 12 months.
Okoro (2022)	Kidney function	Serum creatinine levels at 6 months	↔	μmol/L. Mean(SD); Measured after 6 months.
Okoro (2022)	Kidney function	Serum creatinine levels at 12 months	↔	μmol/L. Mean(SD); Measured after 12 months.
Rifkin (2013)	Kidney function	Creatinine	↔	mg/dl/mean±SD; change - median and IQR. After 6 months.
Alshogran (2022)	Kidney function	Creatinine level	↑	mg/dL; mean±SD. Measured three months later

Bessa (2016)	Kidney function	eGFR	↔	ml/min/1.73m2. mean±SD. Measured at day 90.
Lalonde (2017)	Kidney function	eGFR	↔	mL/min/1.73 m2/mean±SD; unadjusted and adjusted incremental mean change (95% confidence interval). Measured after 12 months.
Rifkin (2013)	Kidney function	eGFR	↔	ml/min/1.73 m2/mean±SD; change - median and IQR. After 6 months.
Tuttle (2018)	Kidney function	eGFR	↔	ml/min/1.73m2/mean±SD. Measured at 30 and 90 days.
Cooney (2015)	Kidney function	Measurement of UACR	↑	No units. N(%); Measured after one year.
Tuttle (2018)	Kidney function	Serum creatinine	↔	mg/dl/mean±SD. Measured at 30 and 90 days.
Tuttle (2018)	Kidney function	UACR	↔	mg/g/median(IQR). Measured at 30 and 90 days.
Al Hamarneh (2018)	Kidney progression	5-year predicted risk for developing ESRD	↔	%; mean±SD, relative reduction and absolute difference (95%CI). Measured after three months.
Theeranut (2021)	Kidney progression	Difference in CKD stage from baseline	↑	No units/n(%) (after three months)
Cooney (2015)	Kidney progression	Incidence of ESRD	↔	No units. N(%); Measured after one year.
Ishani (2016)	Kidney progression	incidence of ESRD	↔	No units/ n(%) (HR(95% CI)). After one year.
Theeranut (2021)	Kidney progression	Mean difference of eGFR from baseline	↑	mL/min/1.73 m2/mean(SD) (after three months)
Theeranut (2021)	Kidney progression	proportion of patients with eGFR decline greater than 4 mL/min/1.73 m2	↑	No units/n(%) (after three months)

Chang (2016)	Lipid screening	Lipid screening within 1 year of the enrolment date	↔	No units/n(%, OR, 95% CI). Measured at end of trial (end of year).
Qudah (2016)	Medication adherence	adherence to antihypertensive therapy	↔	No units/(%). Measured after three months.
Okoro (2022)	Medication adherence	Antihypertensive medication adherence at 6 months	↔	No units. Mean(SD); Measured after 6 months,
Okoro (2022)	Medication adherence	Antihypertensive medication adherence at 12 months	↔	No units. Mean(SD); Measured after 12 months.
Skoutakis (1978)	Medication adherence	Drug compliance (medication adherence)	↑	%/mean±SD (N/R when it was exactly measured).
Chisholm-Burns (2013)	Medication adherence	Immunosuppressant adherence (assessed for medication adherence)	↑	No units/t-test (df); measured at 6 months, 9 months, 12 months, over 1-year study period.3-months followup post-intervention period
Chisholm (2001)	Medication adherence	Immunosuppressant compliance rates (assessed for medication adherence)	↑	%/mean±SD (calculated for each of the 12 months and for the entire 12-month study period
Bessa (2016)	Medication adherence	Medication adherence	↔	No units. n(%). Measured on days 28 and 90
Cooney (2015)	Medication adherence	Medication adherence	↔	No units. Mean(SD); Measured after one year.
Rifkin (2013)	Medication adherence	Medication adherence	↔	No units/mean±SD; change - median and IQR. After 6 months.
Alshogran (2022)	Medication adherence	Medication adherence	↑	No units; mean±SD. Measured three months later.
Song (2021)	Medication adherence	medication adherence for discharge drugs	↔	No units/mean±SD; n(%); time point not stated.
Mateti (2018)	Medication adherence	Medication adherence rate	↑ **for all hospitals**	No units/mean±SD; Greenhouse-Geisser F. Measured at 6th and 12 month.

Yokum (2008)	Medication changes	medication adjustments	↑	No units, median (range). Time when dose adjustments made not specified.
Al Hamarneh (2018)	Medication changes	Medication changes/no. of patients for diabetes	↑	No units; n(%). Measured after three months.
Al Hamarneh (2018)	Medication changes	Medication changes/no. of patients for dyslipidemia	↑	No units; n(%). Measured after three months.
Al Hamarneh (2018)	Medication changes	Medication changes/no. of patients for hypertension	↔	No units; n(%). Measured after three months.
Santschi (2011)	Medication class	Class of antihypertensive medications used	↔	n%). Measured at six months.
Bessa (2016)	Medication discontinuation	Discontinuation of immunosuppressive treatment	↔	No units. n(%). Measured after 90 days.
Song (2021)	Medication discrepancy	Change in the number of unintentional medication discrepancies at discharge compared with that at the time of admission	↑	No units/n(%); time point not stated.
Van den Oever (2020)	Medication dose	Iron sucrose dose	↑	mg/week/median (IQR). End of three months.
Van den Oever (2020)	Medication dose	Patients with mean dose of ≥90 mcg DA per week	↔	No units/n(%). End of three months.
Van den Oever (2020)	Medication dose	Weekly DA dose	↑	mcg/week/median (IQR). End of three months.
Gonzales (2021)	Medication error	Incidence of medication errors	↑	No units. Unadjusted - total (N); mean±SD; adjusted - mean (95%CI), IRR (95% CI) .After 12 months.
Cohen (2020)	Medication error	% of patients with inadequate medication reconciliation determined by any one error in medication reconciliation	↑	No units/n (%). Time point not reported when outcomes were measured.
Bhardwaja (2011)	Medication error	Proportion of medication errors	↑	No units/n(%, 95% CI) and n(%). Measured over 15 months
Gonzales (2021)	Medication error	severity of medication errors	↑	No units. Total (N). After 12 months.

Cohen (2020)	Medication error	The number of medication errors, of all medications and high-risk medications, identified per patient sample	**↑ (no p-value)**	No units/n. Time point not reported when outcomes were measured.
Peralta (2020)	Medication initiation	ACEi/ARB initiation	↔	For as-treated and intention to treat analyses: No units/n(%). After 12 months.
Peralta (2020)	Medication initiation	diuretic initiation	↔	For as-treated and intention to treat analyses: No units/n(%). After 12 months.
Al Hamarneh (2018)	Medication initiation	Initiation of ACEI	↑	No units; n(%). Measured after three months.
Al Hamarneh (2018)	Medication initiation	Initiation of ARB	↔	No units; n(%). Measured after three months.
Al Hamarneh (2018)	Medication initiation	Initiation of statins	↔	No units; n(%). Measured after three months.
Peralta (2020)	Medication initiation	statin therapy initiation	↔	For as-treated and intention to treat analyses: No units/n(%). After 12 months.
Peralta (2020)	Medication use	ACEi/ARB use	↔	For as-treated and intention to treat analyses: No units/n(%). After 12 months.
Peralta (2020)	Medication use	diuretic use	↔	For as-treated and intention to treat analyses: No units/n(%). After 12 months.
Chang (2016)	Medication use	Proportion of patients on statins	↔	No units/n(%, OR, 95% CI). Measured at end of trial (end of year).
Chang (2016)	Medication use	Proportion of proteinuric CKD patients taking an ACEI or ARB	↔	No units/n(%). Measured at end of trial (end of year).
Peralta (2020)	Medication use	statin therapy use	↔	For as-treated and intention to treat analyses: No units/n(%). After 12 months.
Tuttle (2018)	Medication use	use rates of ACE inhibitors or ARBs	↔	No units/n(%). Measured at 30 and 90 days.

Van den Oever (2020)	Mortality	all-cause mortality	↔	No units/%. During follow-up.
Bessa (2016)	Mortality	Death	↔	No units. n(%). Measured after 90 days.
Cooney (2015)	Mortality	Death	↔	No units. N(%); Measured after one year.
Ishani (2016)	Mortality	death	↔	No units/ n(%) (HR(95% CI)). After one year.
Mateti (2018)	Mortality	Survival time	**↔ for all hospitals**	Days/mean(95% CI). No specific time point it was measured at.
Santschi (2011)	Number of medications	Number of antihypertensive drugs	↔	Mean (range) and n(%). Measured at six months.
Rifkin (2013)	Number of medications	Number of blood pressure medications	↔	No units/mean±SD; change - median and IQR. After 6 months.
Cypes (2021)	Number of medications	Number of medications requiring pharmacist intervention	↑	No units/n(%), over a three month period.
Pai (2009)	Number of medications	Number of medications used	↑	No units/mean±SD (measured at end of study).
Cooney (2015)	Number of medications	The number of antihypertensive medications prescribed to those with poorly controlled hypertension	↑	No units. N(%); Measured after one year.
Rifkin (2013)	Number of medications	Total number of medications	↔	No units/mean±SD; change - median and IQR. After 6 months.
Van den Oever (2020)	number of patients with at least one transfusion during follow-up	number of patients with at least one transfusion during follow-up	↑	No units/ n(%). During follow-up.
Skoutakis (1978)	patients' biochemical and therapeutic responses	patients' biochemical and therapeutic responses	↑	No units/n (N/R when it was exactly measured).
Chang (2016)	Proteinuria screening	Proteinuria screening within 1 year of the enrolment date	**Entire population: ↔ Adjusting for age in previously unscreened population: ↑**	No units/n(%, OR, 95% CI). Measured at end of trial (end of year).
Al Hamarneh (2018)	Smoking cessation	Smoking cessation	↑	No units; %, difference in change (95% CI). Measured after three months.

Bessa (2016)	Tacrolimus intrapatient variability	%CV	↔	No units, mean±SD. Measured at days 10, 14, 21, 28, 60, and 90
Bessa (2016)	Tacrolimus intrapatient variability	Mean %CV	↔	No units, mean±SD. Measured at days 7, 10, 14, 21, 28, 60, and 90
Fleming (2021)	Tacrolimus intrapatient variability	Mean TAC intrapatient variability from baseline to 12 months post randomization	↑	(CV) ([mean/SD] · 100) was assessed at monthly intervals for each patient (12-month rolling average)
Fleming (2021)	Tacrolimus intrapatient variability	proportion of patients achieving TAC intrapatient variability of <30% at end of study	↑	No units/% used, but exact figures not provided, as it is displayed on a graph. Measured at month 12.
Fleming (2021)	Tacrolimus intrapatient variability	proportion of patients achieving TAC intrapatient variability of <40% at end of study	↔	No units/% used, but exact figures not provided, as it is displayed on a graph. Measured at month 12.

Table S7 - *Quality assessment of studies*

Author (year)	Criteria number 1	2	3	4	5	6	7	8	9	10	11	12	13	14	Rating
Al Hamarneh (2018)	Yes	Yes	Yes	No for both	No	Yes	Yes	Yes	Yes	Yes	Yes	Yes	Yes	Yes	Good
Alshogran (2022)	Yes	Yes	CD	No for both	NR	No	Yes	Yes	Yes	Yes	Yes	Yes	Yes	No	Poor
Armstrong (2000)	Yes	NR	NR	No for both	NR	Yes	CD	CD	Yes	Yes	Yes	No	Yes	No	Poor
Bessa (2016)	Yes	Yes	Yes	No for participants, CD for providers	No	Yes	Yes	Yes	Yes	Yes	Yes	Yes	Yes	No	Poor
Bhardwaja (2011)	Yes	Yes	CD	Yes for both	Yes	Yes	NR	NR	NR	Yes	Yes	No	Yes	Yes	Fair
Chang (2011)	Yes	Yes	NR	No for both	NR	Yes	No	No	No	Yes	Yes	Yes	Yes	No	Poor
Chisholm-Burns (2013)	Yes	Yes	NR	No for participants, yes for providers	CD	Yes	Yes	Yes	Yes	Yes	Yes	Yes	Yes	Yes	Good
Chisholm (2001)	Yes	NR	NR	No for both	NR	NR	NR	NR	Yes	Yes	Yes	No	Yes	No	Poor
Chisholm (2002)	Yes	NR	NR	No for both	NR	Yes	Yes	Yes	Yes	Yes	Yes	No	Yes	No	Poor
Cohen (2020)	Yes	CD	NR	No for both	NR	No	Yes	Yes	Yes	Yes	Yes	No	Yes	No	Poor
Cooney (2015)	Yes	Yes	Yes	No for both	Yes	Yes	Yes	Yes	Yes	Yes	Yes	Yes	Yes	Yes	Good
Cypes (2021)	Yes	Yes	NR	No for both	CD	Yes	Yes	Yes	Yes	Yes	Yes	No	Yes	No	Poor
Dashti-Khavidaki (2013)	Yes	No	No	No for both	CD	No	No	Yes	No	Yes	Yes	No	Yes	No	Poor
Fleming (2021)	Yes	Yes	NR	No for participants, CD for providers	Yes	Yes	Yes	Yes	Yes	Yes	Yes	Yes	Yes	Yes	Good
Gonzales (2021)	Yes	Yes	NR	No for participants, CD for providers	Yes	Yes	Yes	Yes	Yes	Yes	Yes	Yes	Yes	Yes	Good
Ishani (2016)	Yes	Yes	Yes	No for both	Yes	No	Yes	Yes	Yes	Yes	Yes	Yes	Yes	Yes	Fair
Lalonde (2017)	Yes	Yes	Yes	No for both	CD	Yes	Yes	Yes	Yes	Yes	Yes	Yes	Yes	Yes	Good
Marouf (2020)	Yes	Yes	NR	CD for both	NR	Yes	Yes	Yes	Yes	Yes	Yes	No	Yes	No	Poor
Mateti (2017)	Yes	Yes	Yes	No for both	NR	Yes	No	Yes	No	Yes	Yes	Yes	Yes	No	Poor
Mateti (2018)	Yes	Yes	Yes	No for both	NR	Yes	No	Yes	No	Yes	Yes	Yes	Yes	No	Poor
Mateti (2018)	Yes	Yes	Yes	No for both	NR	Yes	No	Yes	No	Yes	Yes	Yes	Yes	No	Poor
Okoro (2022)	Yes	Yes	Yes	CD for participants, no for provider	Yes	No	No	Yes	No	No	Yes	Yes	Yes	Yes	Fair
Pai (2009)	Yes	Yes	Yes	No for both	NR	Yes	No	Yes	No	Yes	Yes	Yes	Yes	No	Poor
Pai (2009)	Yes	Yes	Yes	No for both	NR	Yes	No	Yes	No	Yes	Yes	Yes	Yes	No	Poor
Peralta (2020)	Yes	Yes	Yes	No for both	Yes	Yes	Yes	Yes	Yes	Yes	Yes	Yes	Yes	Yes	Good
Qudah (2016)	Yes	Yes	No	No for both	NR	Yes	Yes	Yes	Yes	Yes	Yes	No	Yes	No	Poor
Quintana-Barcena (2018)	Yes	Yes	Yes	No for both	NR	Yes	Yes	Yes	Yes	Yes	Yes	No	Yes	Yes	Good
Rifkin (2013)	Yes	No	Yes	No for both	NR	No	Yes	Yes	Yes	No	Yes	Yes	Yes	No	Poor
Santschi (2011)	Yes	Yes	Yes	No for both	NR	Yes	Yes	Yes	Yes	Yes	Yes	No	Yes	Yes	Good
Sathvik (2007)	Yes	Yes	NR	No for both	NR	Yes	CD	CD	Yes	Yes	Yes	No	Yes	No	Poor
Skoutakis (1978)	Yes	Yes	Yes	CD for both	CD	CD	CD	CD	Yes	Yes	Yes	No	Yes	No	Poor
Song (2021)	Yes	Yes	Yes	No for both	Yes	Yes	Yes	Yes	Yes	Yes	Yes	Yes	Yes	No	Poor
Taber (2021)	Yes	Yes	NR	No for patients, CD for providers	Yes	Yes	Yes	Yes	Yes	Yes	Yes	Yes	Yes	Yes	Good
Tamilselvan (2021)	Yes	CD	NR	No for both	NR	CD	Yes	Yes	Yes	Yes	Yes	No	Yes	No	Poor
Theeranut (2021)	Yes	CD	NR	No for both	NR	No	Yes	Yes	Yes	Yes	Yes	Yes	No	No	Poor
Tuttle (2018)	Yes	Yes	Yes	No for both	NR	Yes	No	Yes	No	Yes	Yes	Yes	Yes	Yes	Fair
Van den Oever (2020)	Yes	Yes	Yes	Yes for patients, no for providers	NR	No	Yes	Yes	Yes	Yes	Yes	Yes	Yes	Yes	Fair
Yokum (2008)	Yes	Yes	NR	No for both	NR	Yes	No	No	No	Yes	Yes	Yes	Yes	No	Poor

Abbreviations: CD: Cannot determine; NR: Not reported

Study or Subgroup	Intervention Mean	Intervention SD	Intervention Total	Control Mean	Control SD	Control Total	Weight	Mean difference IV, Random, 95% CI
Al Hamameh 2018	127.7	15.2	147	136.7	19.4	143	13.7%	-9.00 [-13.02 , -4.98]
Chisholm 2002	145.3	16.8	13	175.8	33.9	10	1.0%	-30.50 [-53.41 , -7.59]
Lalonde 2017	134.4	17.7	304	136.1	18.3	138	14.7%	-1.70 [-5.34 , 1.94]
Mateti (Academic hospital) 2018	155.7	7.6	42	160.2	7.1	41	16.0%	-4.50 [-7.66 , -1.34]
Mateti (Corporate hospital) 2018	152.8	6.9	27	162.2	5.3	25	15.5%	-9.40 [-12.73 , -6.07]
Mateti (Government hospital) 2018	160	5.8	9	164	7.4	9	9.0%	-4.00 [-10.14 , 2.14]
Okoro 2022	135.9	17.8	73	140	24.9	74	7.7%	-4.10 [-11.09 , 2.89]
Qudah 2016	137.1	9.4	27	148.4	21.4	25	5.2%	-11.30 [-20.41 , -2.19]
Rifkin 2013	136	15.6	28	140	14.4	15	5.1%	-4.00 [-13.30 , 5.30]
Santschi 2011	135.1	22.2	48	141.8	23.7	41	4.8%	-6.70 [-16.30 , 2.90]
Tuttle 2018	133	21	66	137	21	63	7.3%	-4.00 [-11.25 , 3.25]
**Total (95% CI)**			**784**			**584**	**100.0%**	**-6.06 [-8.45 , -3.67]**

Heterogeneity: $Tau^2 = 6.66$; $Chi^2 = 19.23$, df = 10 (P = 0.04); $I^2 = 48\%$
Test for overall effect: Z = 4.98 (P < 0.00001)
Test for subgroup differences: Not applicable

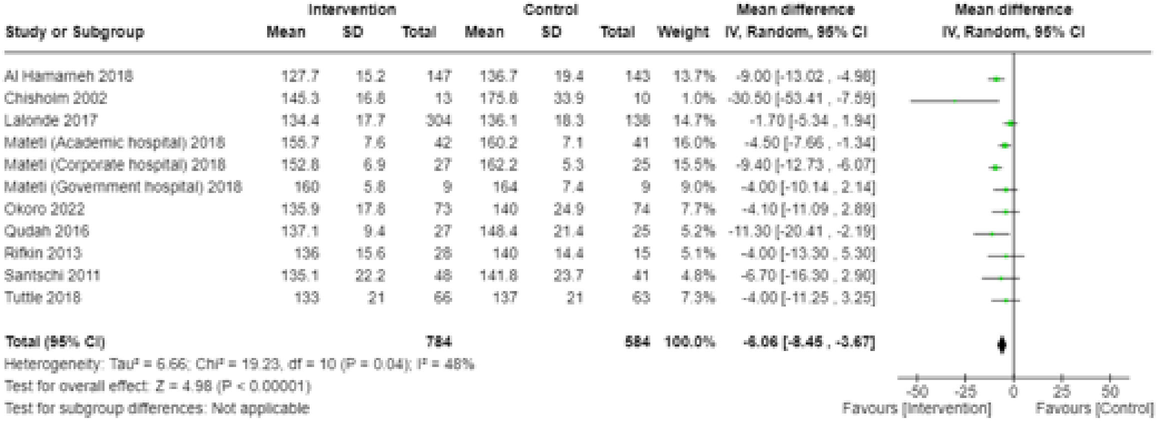

A) Systolic blood pressure

Study or Subgroup	Intervention Mean	Intervention SD	Intervention Total	Control Mean	Control SD	Control Total	Weight	Mean difference IV, Random, 95% CI
Al Hamameh 2018	75.5	11	147	77.7	12.1	143	12.2%	-2.20 [-4.86 , 0.46]
Chisholm 2002	77	10.2	13	91.8	12	10	3.3%	-14.80 [-24.08 , -5.52]
Lalonde 2017	69	10.2	304	69.6	10.5	138	13.4%	-0.60 [-2.69 , 1.49]
Mateti (Academic hospital) 2018	87	4.8	42	91.9	5.8	41	13.0%	-4.90 [-7.19 , -2.61]
Mateti (Corporate hospital) 2018	85.8	5.1	27	92.2	4.6	25	12.2%	-6.40 [-9.04 , -3.76]
Mateti (Government hospital) 2018	90.2	4.9	9	95.1	5	9	8.3%	-4.90 [-9.47 , -0.33]
Okoro 2022	84.4	10.3	73	84.8	14.2	74	9.3%	-0.40 [-4.41 , 3.61]
Qudah 2016	78.2	6.9	27	83.9	10.2	25	7.9%	-5.70 [-10.47 , -0.93]
Rifkin 2013	73	10.3	28	73	12.6	15	4.6%	0.00 [-7.43 , 7.43]
Santschi 2011	67.7	12.5	48	68.8	12.8	41	7.1%	-1.10 [-6.38 , 4.18]
Tuttle 2018	75	14	66	74	11	63	8.7%	1.00 [-3.33 , 5.33]
**Total (95% CI)**			**784**			**584**	**100.0%**	**-3.14 [-5.03 , -1.25]**

Heterogeneity: $Tau^2 = 5.81$; $Chi^2 = 28.53$, df = 10 (P = 0.001); $I^2 = 65\%$
Test for overall effect: Z = 3.25 (P = 0.001)
Test for subgroup differences: Not applicable

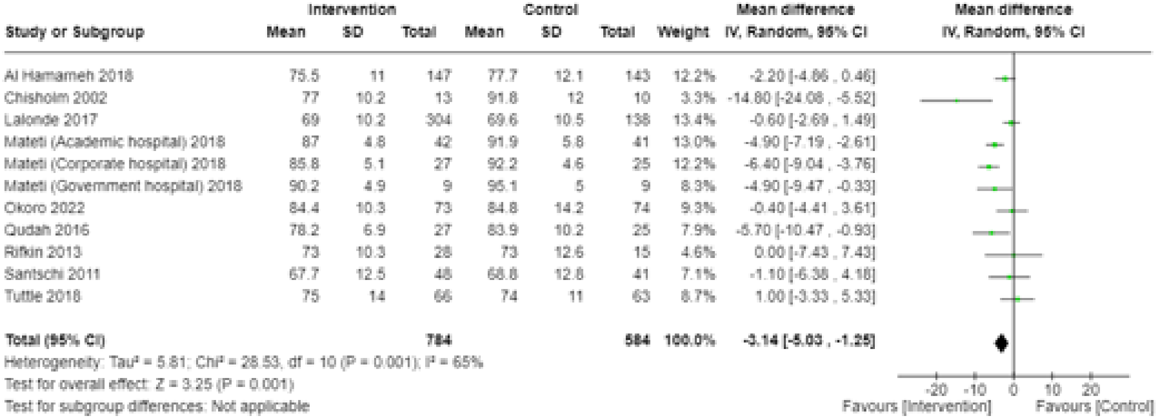

B) Diastolic blood pressure

Figure S2 – Forest plots for systolic blood pressure (S2A) and diastolic blood pressure (S2B)

Study or Subgroup	Intervention Mean	Intervention SD	Intervention Total	Control Mean	Control SD	Control Total	Weight	Std. mean difference IV, Random, 95% CI
Alshogran 2022	8.4	4	57	10.6	3.6	57	26.6%	-0.57 [-0.95 , -0.20]
Okoro 2022	291.6	141.6	73	315.8	134.2	74	29.5%	-0.17 [-0.50 , 0.15]
Rifkin 2013	2.2	0.8	28	2.3	0.8	15	15.7%	-0.12 [-0.75 , 0.51]
Tuttle 2018	1.7	0.7	66	1.6	0.8	63	28.2%	0.13 [-0.21 , 0.48]
**Total (95% CI)**			**224**			**209**	**100.0%**	**-0.19 [-0.50 , 0.13]**

Heterogeneity: Tau² = 0.06; Chi² = 7.42, df = 3 (P = 0.06); I² = 60%
Test for overall effect: Z = 1.17 (P = 0.24)
Test for subgroup differences: Not applicable

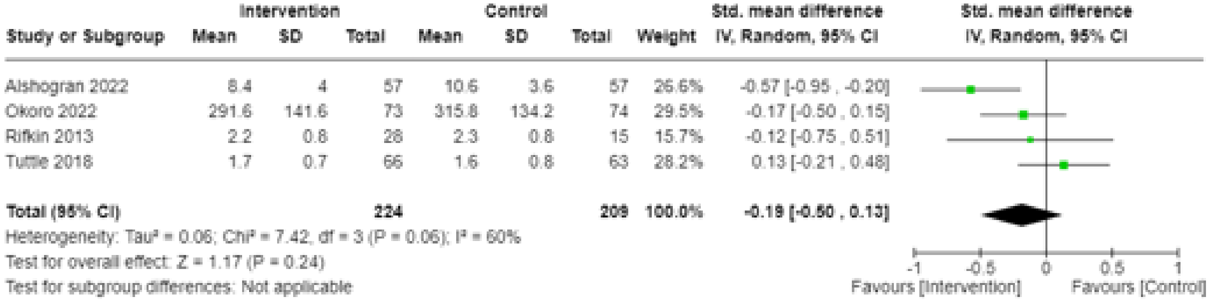

A) Creatinine

Study or Subgroup	Intervention Mean	Intervention SD	Intervention Total	Control Mean	Control SD	Control Total	Weight	Mean difference IV, Random, 95% CI
Alshogran 2022	10.5	1.1	57	9.8	1.3	57	27.4%	0.70 [0.26 , 1.14]
Marouf 2020	11.3	2.3	60	10	2.5	60	12.4%	1.30 [0.44 , 2.16]
Mateti (Academic hospital) 2018	9.8	1.5	42	9	1.5	41	18.4%	0.80 [0.15 , 1.45]
Mateti (Corporate hospital) 2018	10.1	1	27	10.1	1.2	25	19.9%	0.00 [-0.60 , 0.60]
Mateti (Government hospital) 2018	8.4	0.6	9	7.8	0.6	9	21.9%	0.60 [0.05 , 1.15]
**Total (95% CI)**			**195**			**192**	**100.0%**	**0.63 [0.28 , 0.98]**

Heterogeneity: Tau² = 0.07; Chi² = 6.90, df = 4 (P = 0.14); I² = 42%
Test for overall effect: Z = 3.53 (P = 0.0004)
Test for subgroup differences: Not applicable

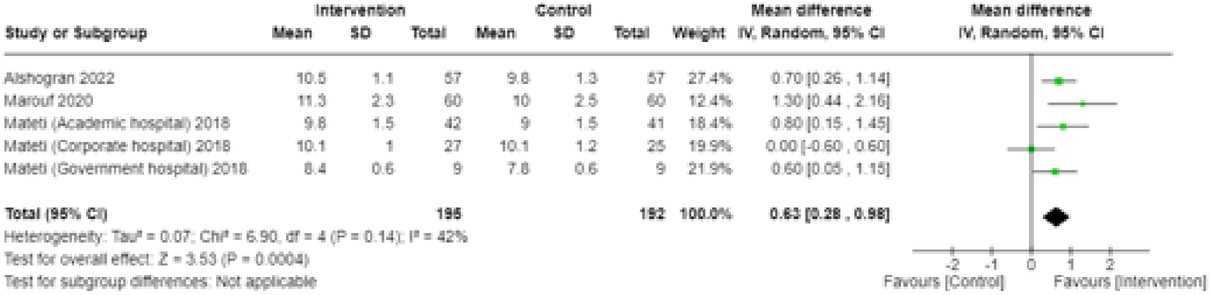

B) Hemoglobin

Figure S3 - Forest plots for creatinine (S3A) and hemoglobin (S3B)
